# Supplementary material for: Impact of scaling up dolutegravir on antiretroviral resistance in South Africa: A modeling study
Source: PLoS Med. 2020 Dec 14;17(12):e1003397. doi: 10.1371/journal.pmed.1003397 (PMC7735592; doi:10.1371/journal.pmed.1003397)
Supplement: S1 Text — Section 1: description of the adapted MARISA model. Section 2: description of model parameters. Section 3: model simulation. Section 4: model equations. Section 5: additional results and sensitivity analyses. (PDF) [file pmed.1003397.s001.pdf]

Supplementary material

Impact of scaling up dolutegravir on antiretroviral resistance in South Africa: A modeling study

Anthony Hauser[1], Katharina Kusejko[2], Leigh Johnson[3], Huldrych Günthard[2,4], Julien Riou[1], Gilles Wandeler[1,5], Matthias Egger[1,3,6,*], and Roger Kouyos[2,4,*]

[1]Institute of Social and Preventive Medicine, University of Bern, Switzerland
[2]Division of Infectious Diseases and Hospital Epidemiology, University Hospital Zurich, University of Zurich, Zurich, Switzerland
[3]Centre for Infectious Disease Epidemiology and Research, University of Cape Town, South Africa
[4]Institute of Medical Virology, University of Zurich, Zurich, Switzerland
[5]Department of Infectious Diseases, Bern University Hospital, University of Bern, Bern, Switzerland
[6]Population Health Sciences, Bristol Medical School, University of Bristol, Bristol, United Kingdom
[*]Corresponding authors (matthias.egger@ispm.unibe.ch, roger.kouyos@uzh.ch)

December 4, 2020

Contents

1 Adapted MARISA model **2**
1.1 MARISA model . . . 2
1.2 Adapted MARISA model . . . 2

2 Parameters and rates of the adapted MARISA model **3**
2.1 Rates related to continuum of care and disease progression . . . 3
2.2 Diagnosis, treatment initiation and switching rates . . . 5
2.3 Resistance rates . . . 5
2.4 Impact of NNRTI-resistance on NNRTI . . . 6
2.5 DTG-efficacy and impact of NRTI-resistance on DTG . . . 7
2.6 Other parameters: HIV transmission and mortality . . . 8

3 Model simulation **9**
3.1 Prospective scenarios . . . 9
3.2 Sensitivity analysis . . . 9

4 Model ODEs **10**
4.1 Description of the compartments . . . 10
4.2 Model ODEs . . . 11

5 Sensitivity analysis and additional results **13**
5.1 Effect of no Treat-All policy . . . 15
5.2 Effect of treatment interruption . . . 17
5.3 Effect of NRTI-resistance and higher efficacy of DTG . . . 18

1 Adapted MARISA model

1.1 MARISA model

MARISA is a mechanistic, compartmental model developed to capture the dynamics of HIV NNRTI resistance among adults in South Africa over the years 2005-2016. It models the continuum of care - including NNRTI-based first-line and PI-based second-line regimens -, the disease progression, acquisition and transmission of NNRTI-resistance, and its impact on the efficacy of NNRTI-based regimen. The model was calibrated using different sources of data: 1) cohort data about more than 30,000 people living with HIV from IeDEA collaboration [1], 2) data from literature, and 3) general HIV estimates at the country scale produced by the Thembisa model. The Thembisa model is a compartmental model providing UNAIDS with estimates on the South African HIV epidemic [2].
We adapted this model to investigate the impact of the introduction of DTG-based regimens in South Africa from 2020. The changes include 1) incorporating DTG-based regimen into the continuum of care, 2) distinguishing between DTG-eligible and -ineligible individuals, and 3) adding a NRTI-resistance dimension.

1.2 Adapted MARISA model

The adapted MARISA model is split in 5 dimensions: 1) care stages (15 levels), 2) disease progression, characterised by the CD4 counts (4 levels), 3) sex (2 levels), 4) NNRTI resistance (2 levels) and 5) NRTI resistance (2 levels).
The first dimension of the model accounts for the whole continuum of care (see Fig A). The first three compartments model respectively HIV-infection of susceptible individuals and diagnosis (with a distinction between DTG-eligible and -ineligible women). We then considered the three different regimens - NNRTI-based, PI-based and DTG-based -, again with a distinction by DTG-eligibility for individuals on a NNRTI-based regimen. For each of the three regimens, three compartments are used to model treatment initiation ("Treat init.") with subsequent virological suppression ("Supp") or failure ("Fail"). "Treat init." compartments represent individuals who initiated treatment less than 3 months ago. Before 2020, all individuals receive a NNRTI-based first-line regimen and switch to the second-line PI-based regimen in case of prolonged failure. From 2020, the DTG-based regimen is used as a first-line regimen for all DTG-eligible individuals. From this time, DTG-eligible individuals who are currently on NNRTI-based regimen can transition to DTG-based regimen. PI-based regimen is still used as a second-line regimen, either for DTG-ineligible patients failing NNRTI-based ART, or for patients failing DTG-based ART.
The second dimension splits individuals in 4 classes according to CD4 counts: 1) $CD4 > 500$ cells/μL, 2) $350 < CD4 < 500$ cells/μL, 3) $200 < CD4 < 350$ cells/μL and 4) $CD4 < 200$ cells/μL. The third dimension makes the distinction between male and female. The fourth and fifth dimensions respectively model NNRTI- and NRTI-resistance. They each have two layers that distinguish between NNRTI-/NRTI-susceptible and -resistant individuals. We used the following indices to indicate a layer of a dimension: j for the second dimension ($j = 1, 2, 3, 4$), k for the third dimension ($k = 1, 2$), l for the fourth dimension ($l = 1, 2$) and m for the fifth dimension ($m = 1, 2$).

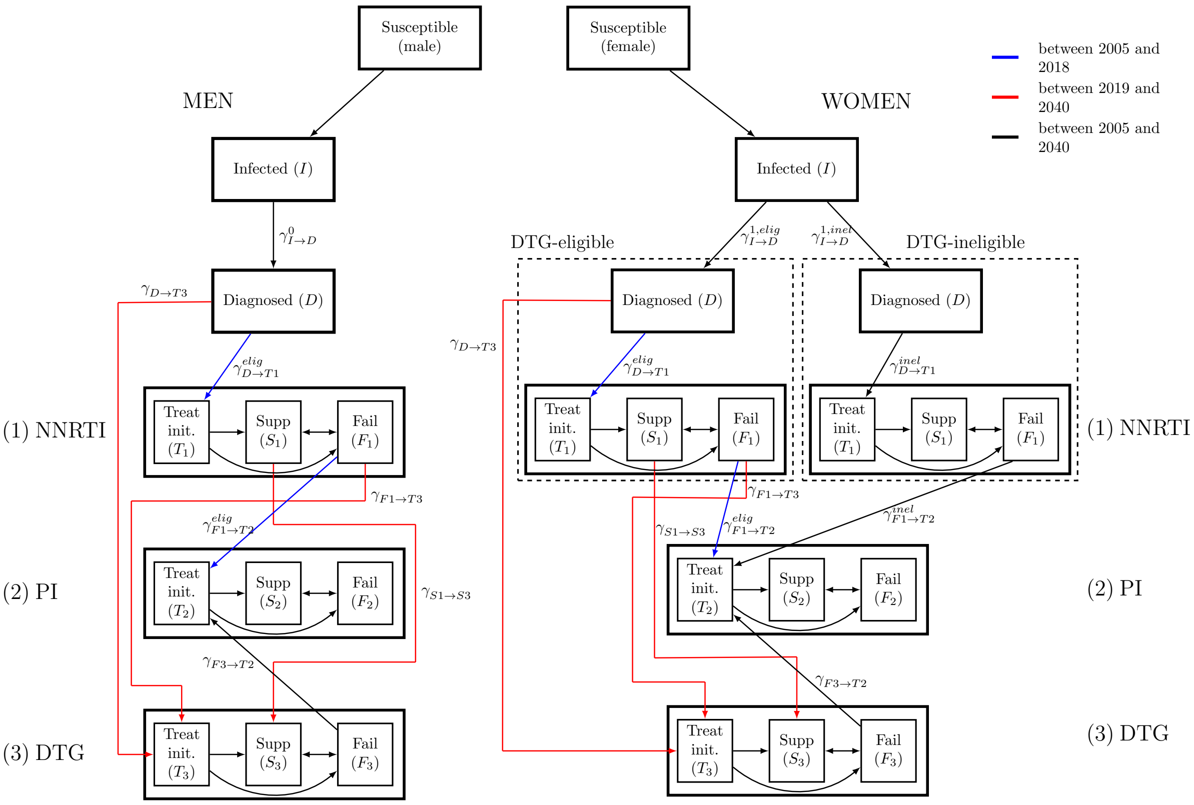

Fig A: Adapted MARISA model. Only the first (continuum of care) and the third (sex) dimensions are represented.

2 Parameters and rates of the adapted MARISA model

2.1 Rates related to continuum of care and disease progression

Rates related to disease progression ν_{CD4} and $\tilde{\nu}_{CD4}$ as well as rates related to continuum of care γ, which respectively model transition from one to another CD4 class and transition from one to another care stage, were estimated using observational cohort data from IeDEA-SA collaboration. Survival analyses were performed using information of more than 30'000 patients from South Africa. Mean estimates and 95% confidence intervals (95%CI) are reported in Table A and B.

Table A: Rates related to disease progression. Rates are in month^{-1}.

Parameter	Description	Values [95% CI]		
Parameters related to disease progression				
		CD4 class		
		$1 \to 2$	$2 \to 3$	$3 \to 4$
$1/\nu_{CD4}^{I}$	Average time to progress from one to another CD4 class, at I (taken from [3])	60	36	42
$1/\nu_{CD4}^{D}$	Average time to progress from one to another CD4 class, at D (taken from [3])	60	36	42
$1/\nu_{CD4}^{T_1}$	Average time to progress from one to another CD4 class, at T_1	47 [42,54]	30 [28,34]	60 [55,66]
$1/\nu_{CD4}^{F_1}$	Average time to progress from one to another CD4 class, at F_1	18 [16,20]	15 [14,16]	22 [21,24]
$1/\nu_{CD4}^{T_2}$	Average time to progress from one to another CD4 class, at T_2	32 [14,72]	22 [12,43]	33 [17,64]
$1/\nu_{CD4}^{F_2}$	Average time to progress from one to another CD4 class, at F_2	14 [8,26]	15 [8,27]	16 [10,25]
		$1 \leftarrow 2$	$2 \leftarrow 3$	$3 \leftarrow 4$
$1/\tilde{\nu}_{CD4}^{T_1}$	Average time to progress from one to another CD4 class, at T_1	16 [15,17]	16 [15,17]	18 [17,19]
$1/\tilde{\nu}_{CD4}^{S_1}$	Average time to progress from one to another CD4 class, at S_1	17 [16,17]	14 [14,14]	9 [9,10]
$1/\tilde{\nu}_{CD4}^{T_2}$	Average time to progress from one to another CD4 class, at T_2	16 [9,27]	19 [11,31]	41 [23,73]
$1/\tilde{\nu}_{CD4}^{S_2}$	Average time to progress from one to another CD4 class, at S_2	17 [13,21]	14 [11,17]	7 [6,10]

Table B: Rates related to transition between care stages. Rates are in month^{-1}.

Parameter	Description	Values [95% CI]			
Parameters related to care stages					
		CD4 class			
		1	2	3	4
$1/\gamma_{T_1 \to S_1}$	Time from T_1 to S_1	3.4 [3.3,3.6]	3.5 [3.3,3.7]	3.6 [3.4,3.7]	3.9 [3.8,4.1]
$1/\gamma_{T_1 \to F_1}$	Time from T_1 to F_1	23.4 [20.1,27.3]	22.8 [19.6,26.4]	18.9 [17.5,20.4]	12.9 [12.3,13.5]
$1/\gamma_{S_1 \to F_1}$	Time from S_1 to F_1	176.3 [157,197.9]	133.8 [118.6,150.8]	62.1 [57,67.6]	22.1 [20.2,23.9]
$1/\gamma_{F_1 \to S_1}$	Time from F_1 to S_1	6.4 [5.5,7.4]	12.9 [11,14.9]	14.3 [12.9,15.9]	18.2 [16.3,20.2]
$1/\gamma_{F_1 \to T_2}$	Time from F_1 to T_2	467.5 [243,898.9]	376 [240.4,589.9]	258.9 [200.7,334.6]	166.4 [140,199]
$1/\gamma_{T_2 \to S_2}$	Time from T_2 to S_2	3.8 [2.7,5.2]	3.8 [2.6,5.5]	4 [3,5.3]	5 [4,6.4]
$1/\gamma_{T_2 \to F_2}$	Time from T_2 to F_2	14.3 [7.8,26.8]	14 [7.3,27]	11.8 [7.8,18]	7.6 [5.9,9.9]
$1/\gamma_{S_2 \to F_2}$	Time from S_2 to F_2	61.4 [30.8,122.8]	40.9 [21.4,78.9]	40 [21.4,74.3]	19.1 [9,40]
$1/\gamma_{F_2 \to S_2}$	Time from F_2 to S_2	2.3 [1.1,4.1]	12.9 [3.2,51.3]	5.5 [2.8,11.3]	11.7 [4.8,28]

2.2 Diagnosis, treatment initiation and switching rates

Diagnosis rates depend on sex and CD4 class and treatment initiation rates depend on CD4 class. They have been described in details in [4], S1 File, Section 1.3. In the adapted MARISA model, we assumed that diagnosis rates are constant from 2016, while the treatment initiation rate has been adapted in order to model the impact of the Treat-All policy. We increased treatment initiation rates for the first three CD4 count classes from 2017 to 2022 in order to have identical rates from 2022, irrespective of CD4 counts (see Fig B). To ensure a proportion p_1 of DTG-eligible women, two diagnosis rates are used $\gamma_{I\to D}^{k,elig} := p_1 \cdot \gamma_{I\to D}^{k}$, $\gamma_{I\to D}^{k,inel} := (1-p_1)\cdot \gamma_{I\to D}^{k}$, in order to distribute women into the two DTG-eligibility classes.

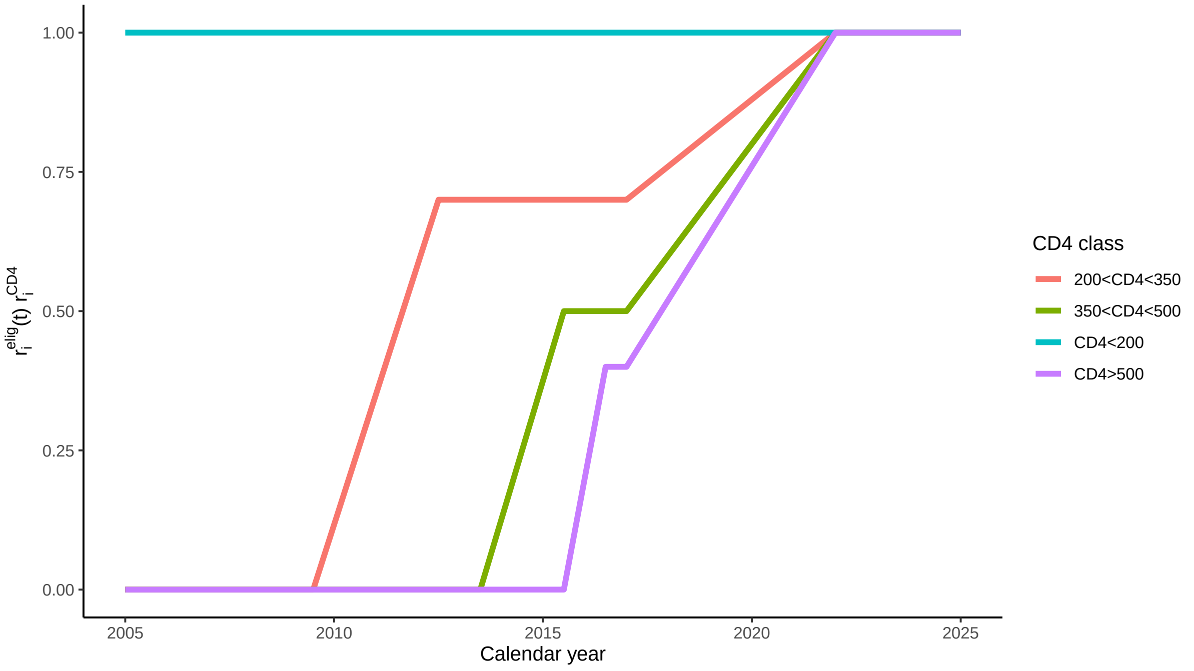

Fig B: $r_i^{elig}(t)\cdot r_i^{CD4}$ represents the level of treatment eligibility $r_i^{elig}(t)$ multiplied by r_i^{CD4}, representing the lower treatment initiation rate of CD4 class i relative to the CD4 class $i=4$. These two components are parts of the overall treatment initiation rate $\gamma_{D\to T_1}^{i}(t) = \gamma_{2005}^{CD4<200}\cdot r_i^{elig}(t)\cdot r_i^{CD4}\cdot r^{time}(t)$.

We rescaled the switching rates from unsuppressed NNRTI-based regimen to PI-based regimen $\gamma_{F_1\to T_2}^{k,elig/inel}$ in order to reflect PI-coverage in South Africa ($\sim 4\%$ in 2016 according to [5]). The new rates $\gamma_{F_1\to T_2}^{k,elig/inel}$ can be found in Table B.

2.3 Resistance rates

Two rates model the flow between the two NNRTI-resistance layers: the reversion rate σ_{rev} and the rate of acquiring NNRTI-resistance σ_{res}^{NNRTI}. Reversion to wild-type occurs when no more drug pressure is exerted, i.e. in the "Infected" and "Diagnosed" compartments. An individual can acquire NNRTI-resistance when failing first-line regimen. Both parameters σ_{rev} and σ_{res}^{NNRTI} were collected from literature and can be found in Table C.

Table C: Parameters collected from literature. As mortality estimates in the fourth CD4 class vary according to the proportion of people with $CD4 < 50$ cells/μL, lower and upper bounds are given (see [4] S1 File Section 1.2 for more details). The mortality risk μ_X^j in CD4 class j ($i = 1, \ldots, 4$) and care stage X ($X = I, D, T_1, \ldots$) is given by: $\mu_X^j = \mu_0 \cdot \tilde{\mu}_X^j$.

Parameter	Description	Values	Ref		
Resistance parameters					
$1/\sigma_{res}^{NNRTI}$	Time to acquire NNRTI-resistance (in month)	5	[6, 7, 8, 9, 10]		
$1/\sigma_{res}^{NRTI}$	Time to acquire NRTI-resistance (in month)	40	[11]		
$1/\sigma_{rev}$	Time to revert back to wild-type (in month)	125	[12]		
α_1	Impact of NNRTI-resistance on NNRTI-based ART	1.97	[13, 14]		
α_2	Impact of NNRTI-resistance on NNRTI-based ART	3.24	[13, 14]		
α_3	Impact of NRTI-resistance on DTG-based ART	1	[15, 16, 17]		
α_4	Scaling factor for the efficacy NNRTI-based regimen (see Section 2.4)	1.62	[1]		
α_5	Scaling factor for the efficacy DTG-based regimen (see Section 2.5)	0.85	[18]		
Other parameters					
$\nu_{0,0}$	probability that a male infects a male (per act)	0.8%	[19]		
$\nu_{0,1}$	probability that a male infects a female (per act)	0.3%	[19]		
$\nu_{1,0}$	probability that a female infects a male (per act)	0.3%	[19]		
$\rho_{0,0}$	percentage of MSM	5%	[20]		
$\tilde{\mu}^i$	relative mortality risk		[21] [22]		
	(Ref: suppressed indiv. with CD4>500)		CD4 class		
		1	2	3	4
	$\tilde{\mu}^i_{I/D}$: not treated (I and D)	1.6	2	4.6	40.9-134.4
	$\tilde{\mu}^i_{T_1/T_2}$: started treatment (T_1 and T_2)	2.5	2.6	3.1	10-50.7
	$\tilde{\mu}^i_{S_1/S_2}$: suppressed (S_1 and S_2)	1	1.3	2	8.3-41.7
	$\tilde{\mu}^i_{F_1/F_2}$: failed (F_1 and F_2)	3.9	3.9	4.3	11.8-59.7

For the sake of simplicity and in view of the scarcity of evidence on the impact of NRTI-resistance on DTG-based regimen, the dimension modelling NRTI-resistance has only two layers that distinguish between NRTI-resistant and NRTI-susceptible individuals. NRTI resistance is defined as having both the K65R and the M184V mutations, which confers high level of resistance to tenofovir (TDF) and lamivudine/emtricitabine (3TC/FTC), respectively. In view of the low level of NRTI pre-treatment drug resistance (PDR) [12, 23], we assume that NRTI resistance is not transmitted. The rate σ_{res}^{NRTI} models the process of acquiring NRTI-resistance, which occurs when individuals are failing first-line NNRTI-based regimen. We calibrated σ_{res}^{NRTI} using results from a meta-analysis that estimates the prevalence of NRTI resistance mutation after 3 years on a failing NNRTI-based first-line regimen [11]. This meta-analysis found that 75% of them had the K65R mutation and 73% the M184V mutation. Assuming no association between the two mutations as suggested by [24], we inferred σ_{res}^{NRTI} so that 54.8% (i.e. 75%·73%) of individuals failing NNRTI-based regimen were resistant to NRTI after 3 years of ART. We found $\sigma_{res}^{NRTI} = 1/40$ months^{-1} (see Table C).

2.4 Impact of NNRTI-resistance on NNRTI

Unlike the previous MARISA model, the adapted MARISA used two parameters α_1 and α_2 to model the impact of NNRTI resistance on NNRTI treatment response. Both parameters increase the previously estimated rates of failure $\gamma_{T_1 \to F_1}$, $\gamma_{S_1 \to F_1}$ and decrease the suppression rates $\gamma_{T_1 \to S_1}$ and $\gamma_{F_1 \to S_1}$ for NNRTI-resistant individuals, but at different treatment stages. While α_1 represents the impact of NNRTI resistance among individuals having just started treatment (less than 3 months), α_2 models this impact at the later stage of treatment. In order that the MARISA model achieves the same suppression level with these modified rates as estimated from IeDEA-SA cohort data, we used a third scaling parameter α_4 which increases the overall suppression rates and decreases the failing rates. The different failing and suppression rates according to CD4 class j and NNRTI-resistance status l are given in Eq 1-8. The rates $\gamma_{T_1 \to F_1}$, $\gamma_{T_1 \to S_1}$, $\gamma_{S_1 \to F_1}$ and $\gamma_{F_1 \to S_1}$ represent the overall suppression and failure rate for NNRTI-based ART, as estimated with IeDEA cohort data (see Tables A and B).

$$\gamma_{T_1 \to F_1}^{j,l=0} = 1/\alpha_4 \cdot \gamma_{T_1 \to F_1} \tag{1}$$

$$\gamma^{j,l=1}_{T_1 \to F_1} = \alpha_1/\alpha_4 \cdot \gamma_{T_1 \to F_1} \tag{2}$$

$$\gamma^{j,l=0}_{T_1 \to S_1} := 1/3 - \gamma^{j,l=0}_{T_1 \to F_1} \tag{3}$$

$$\gamma^{j,l=1}_{T_1 \to S_1} := 1/3 - \gamma^{j,l=1}_{T_1 \to F_1} \tag{4}$$

$$\gamma^{j,l=0}_{F_1 \to S_1} = \alpha_4 \cdot \gamma_{F_1 \to S_1} \tag{5}$$

$$\gamma^{j,l=1}_{F_1 \to S_1} = \alpha_4/\alpha_2 \cdot \gamma_{F_1 \to S_1} \tag{6}$$

$$\gamma^{j,l=0}_{S_1 \to F_1} = 1/\alpha_4 \cdot \gamma_{S_1 \to F_1} \tag{7}$$

$$\gamma^{j,l=1}_{S_1 \to F_1} = \alpha_2/\alpha_4 \cdot \gamma_{S_1 \to F_1} \tag{8}$$

The three parameters α_1, α_2 and α_3 were simultaneously calibrated using two different kinds of data. To identify α_1 and α_2, we used estimates from two studies that compared level of NNRTI failure between NNRTI-susceptible and NNRTI-resistant individuals. Both studies reported a hazard ratio (HR) of ART failure between NNRTI-resistant and NNRTI-susceptible people of 3.13. To identify α_4, we used the overall suppression level of 88% for NNRTI-based regimen, as estimated from IeDEA cohort data. The values of the three parameter estimates are given in Table C. The higher estimated value of α_2 compared with α_1 ($\alpha_1 = 1.97$, $\alpha_2 = 3.24$) reflects the long-term impact of NNRTI-resistance on the NNRTI-treatment response.

2.5 DTG-efficacy and impact of NRTI-resistance on DTG

In this updated MARISA model, we also model the potential impact of NRTI-resistance on DTG-based regimen. We used the same suppression and failure rates for DTG-based regimen as for NNRTI-based one, but replace the scaling factor α_4 by α_5, to take into account the difference in treatment efficacy between NNRTI and DTG. The scaling factor α_5 was calibrated to reflect results of the NAMSAL study [18], which observed a crude odds ratio (OR) of failure of 1.46 between NNRTI- and DTG-based regimens, after 48 weeks of treatment. To do so, we fitted the OR calculated by the MARISA model to the OR observed in NAMSAL studies, taking into account the different baseline characteristics (distribution of CD4 counts and level of baseline NNRTI-resistance) of the NNRTI- and DTG-groups. After these adjustments, we found an OR of 1.02 between the two groups, assuming that they are both susceptible to their respective ART regimen (i.e. no NNRTI-resistance). This decrease in OR after adjustment is due to the fact that the NNRTI-group in the NAMSAL had lower baseline CD4 counts and that part of them had baseline NNRTI-resistance. Other efficacies of DTG-based regimens, corresponding to ORs of 2 and 5, were investigated in the sensitivity analysis (see Section 5).
As a simplifying assumption, all individuals that transitions to DTG-based regimen are considered to have received a NNRTI-drug combined with TDF and 3TC/FTC and to keep this NRTI-backbones combination after transitioning to DTG-based regimen. This assumption is motivated by the expected reluctance of clinicians to prescribe zidovudine (AZT) for TDF-experienced individuals transitioning to DTG due to its side effects. In the case where NRTI backbones would be adapted when transitioning to DTG, the model might overestimate the impact of NRTI resistance on DTG-based regimen. We applied the same approach to model the impact of NRTI resistance on DTG-based regimen as we did to model the impact of NNRTI resistance on NNRTI-based regimen. The suppression and failure rates for NRTI-resistant individual starting a DTG-based regimen are respectively divided and multiplied by a factor α_3. The different failing and suppression rates according to CD4 class j and NRTI-resistance status m are given in Eq 9-16.

$$\gamma^{j,m=0}_{T_3 \to F_3} = 1/\alpha_5 \cdot \gamma_{T_1 \to F_1} \tag{9}$$

$$\gamma^{j,m=1}_{T_3 \to F_3} = \alpha_3/\alpha_5 \cdot \gamma_{T_1 \to F_1} \tag{10}$$

$$\gamma^{j,m=0}_{T_3 \to S_3} := 1/3 - \gamma^{j,l=0}_{T_1 \to F_1} \tag{11}$$

$$\gamma^{j,m=1}_{T_3 \to S_3} := 1/3 - \gamma^{j,l=1}_{T_1 \to F_1} \tag{12}$$

$$\gamma^{j,m=0}_{F_3 \to S_3} = \alpha_5 \cdot \gamma_{F_1 \to S_1} \tag{13}$$

$$\gamma^{j,m=1}_{F_3 \to S_3} = \alpha_5/\alpha_3 \cdot \gamma_{F_1 \to S_1} \tag{14}$$

$$\gamma^{j,m=0}_{S_3\to F_3} = 1/\alpha_5 \cdot \gamma_{S_1\to F_1} \tag{15}$$

$$\gamma^{j,m=1}_{S_3\to F_3} = \alpha_3/\alpha_5 \cdot \gamma_{S_1\to F_1} \tag{16}$$

In the main analysis, we calibrated α_3 so that the odds ratio (OR) of DTG-failure between NRTI-susceptible and -resistant individuals takes two particular values : OR=1, OR=2. Higher impact of NRTI-resistance on DTG-based regimen (OR=5) is investigated in the sensitivity analysis, together with different DTG-efficacies (see Section 5).

2.6 Other parameters: HIV transmission and mortality

The MARISA model accounts for both heterosexual and homosexual HIV-transmission, with different risks of transmission per intercourse. We also assumed that undiagnosed individuals have a more risky behaviour. Parameters related to HIV-transmission were either collected from literature (see Table C) or estimated using results from Thembisa model (see Table D). We assumed that mortality depends on both the CD4 counts and the treatment stage. Relative mortality estimates were collected from literature (see Table C) and a scaling parameter, representing the mortality risk among suppressed invidual with CD4>500 copies/ml, was fitted to HIV-mortality estimate provided by the Thembisa model. More information about HIV-transmission and mortality can be found in S1 Text of [4].

Table D: Parameters estimated from outputs of the Thembisa model.

Parameter	Description	Values
β_u	Number of unprotected sexual acts per month (for undiagnosed individual)	3.1
β_d	Number of unprotected sexual acts per month (for diagnosed individual)	1.24
$\gamma_{I\to D}(2016)/\gamma_{I\to D}(2005)$	Ratio of diagnosis rates between 2005 and 2016	4.4
$1/\gamma_{I\to D}(2005)$	Time to diagnosis in 2005 (in month)	26
$1/\gamma_{D\to T_1}(2005)$	Time to ART initiation in 2005 (in month)	60
μ_0	Mortality risk (in $(\text{month}\cdot 1000\ \text{people})^{-1}$) for a suppressed individual with $CD4 > 500$ cells/μL	0.08

3 Model simulation

3.1 Prospective scenarios

We simulated 2 different scenarios:

1. DTG only used in first-line regimen of ART-initiators and, as second-line, in patients failing NNRTI-based ART ($\gamma_{D\to T3}$ and $\gamma_{F1\to T3}$ in Fig A),
2. DTG used as initial first-line regimen (for ART-initiators), with all patients on NNRTI-based regimens being switched to a DTG-based regimen (all the red arrows).

Within these 2 scenarios, 4 sub-scenarios investigated the impact of different percentages p_1 of DTG-prescription for women: a) no women (0%), b) women outside reproductive age (17.5%), c) women outside reproductive age or using contraception (63%), and d) all women (100%). Percentage in b) is calculated with the help of IeDEA-SA cohorts [1] which estimated that 17.5% of adult women under ART were older than 49. Percentage in c) is calculated with the help of both IeDEA-SA estimate and World Bank [25], which estimated that 54.6% of women aged 15-49 in South Africa were using any contraception method in 2015:

$$\begin{aligned} p_1 :=& \mathbb{P}(\text{women eligible for DTG}) \\ =& 1-\mathbb{P}(15 \leq age \leq 49 \text{ \& no contraception}|\text{women on ART}) \\ =& 1-\mathbb{P}(15 \leq age \leq 49|\text{women on ART}) \cdot \mathbb{P}(\text{no contraception}|15 \leq age \leq 49 \text{ \& on ART}) \\ =& 1-(1-0.175)\cdot(1-0.546) = 63\%. \end{aligned} \tag{17}$$

As no information about contraceptive prevalence in South African adult women on ART have been found, we approximated it by the contraceptive prevalence in the general South African adult women population (see 17). By definition, the percentage p_0 of DTG-eligible men is 100%.

3.2 Sensitivity analysis

In the sensitivity analysis, we perturbed eight parameters 200 times using a Latin Hypercube Sampling method (see Table E). Table E displays the main values of the eight parameters, which were informed from literature and lower and upper bounds, chosen to reflect plausible values of the parameters. As varying the transmission-related parameters may modify the overall transmission rate, an adjustment is made to have a transmission rate similar to the baseline model. We ran the sensitivity analysis for each prospective scenario (13 different scenarios in total).

Table E: Parameter ranges used in sensitivity analysis. Lower and upper bounds for α_1 and α_2 were determined in order to have an OR of ART failure between NNRTI-susceptible and -resistant individuals of 1 and 5, respectively. For α_5, lower and upper bounds were determined in order to have an OR between NNRTI- and DTG-failure of 1 and 2, respectively.

Parameter	Definition	Value	Lower bound	Upper bound
Resistance-related parameters				
$1/\sigma_{res}^{NNRTI}$	Time to acquisition of NNRTI resistance (months)	5	3	9
$1/\sigma_{rev}$	Time to reversion to wild-type virus (months)	125	36	200
α_1	Impact of NNRTI resistance on NNRTI-based ART	1.97	1 (OR=1)	3.1 (OR=5)
α_2	Impact of NNRTI resistance on NNRTI-based ART (see Eq 1-8)	3.24	1 (OR=1)	5.1 (OR=5)
α_5	Scaling factor for the efficacy DTG-based ART	0.85	0.84 (OR=1)	1.25 (OR=2)
Transmission-related parameters				
$\rho_{0,0}$	Percentage of MSM	5%	1%	10%
$\nu_{0,0}/\nu_{0,1}$	Increase in risk of transmission in MSM (see Table C	2.7	1	5
-	Ratio between HIV prevalence in MSM and in HET	1	1	3

4 Model ODEs

4.1 Description of the compartments

Table F describes the compartments used in the model, while model ODEs are given in Equations 19.

Table F: Description of the compartments used in the model.

Notation	Description	Definition
Dimensions/Compartments		
j	index for the 2nd dimension (CD4 counts)	$j = 1, 2, 3, 4$ (4 CD4 strata)
k	index for the 3rd dimension (sex)	$k = 0$: men, $k = 1$: women
l	index for the 4th dimension (NNRTI-resistance)	$l = 0$: NNRTI-susceptible<br>$l = 1$: NNRTI-resistant
m	index for the 5th dimension (NRTI-resistance)	$m = 0$: NRTI-susceptible<br>$m = 1$: NRTI-resistant
$I^{jklm}(t)$	number of infected (not diagnosed) indiv.	
$D^{jklm}_{elig}(t)$,<br>$D^{jklm}_{inel}(t)$	number of diagnosed (not treated) indiv.<br>for resp. DTG-eligible and -ineligible ind. (by def. $D^{j0lm}_{inel}(t) = 0$)	
NNRTI-based treatment		
$T^{jklm}_{1,elig}(t)$,<br>$T^{jklm}_{1,inel}(t)$	number of indiv. that have started NNRTI-based treatment for less than 3 months<br>for resp. DTG-eligible and -ineligible ind. (by def. $T^{j0lm}_{1,inel}(t) = 0$)	
$S^{jklm}_{1,elig}(t)$,<br>$S^{jklm}_{1,inel}(t)$	number of suppressed indiv. on NNRTI-based treatment<br>for resp. DTG-eligible and -ineligible ind. (by def. $S^{j0lm}_{1,inel}(t) = 0$)	
$F^{jklm}_{1,elig}(t)$,<br>$F^{jklm}_{1,inel}(t)$	number of indiv. failing NNRTI-based treatment<br>for resp. DTG-eligible and -ineligible ind. (by def. $F^{j0lm}_{1,inel}(t) = 0$)	
PI-based treatment		
$T^{jklm}_{2}(t)$	number of indiv. that have started PI-based treatment for less than 3 months	
$S^{jklm}_{2}(t)$	number of suppressed indiv. on PI-based treatment	
$F^{jklm}_{2}(t)$	number of indiv. failing PI-based treatment	
DTG-based treatment		
$T^{jklm}_{3}(t)$	number of indiv. that have started DTG-based treatment for less than 3 months	
$S^{jklm}_{3}(t)$	number of suppressed indiv. on DTG-based treatment	
$F^{jklm}_{3}(t)$	number of indiv. failing DTG-based treatment	
Aggregated compartments		
$Susc^k$	number of susceptible indiv. of sex k	
$Inf^{kl}_u(t)$	number of undiagnosed indiv.	$Inf^{kl}_u(t) := I^{kl}(t)$
$Inf^{kl}_d(t)$	number of infectious diagnosed indiv.	

4.2 Model ODEs

The rates γ represent the transition between care stages, ν_{CD4} the transition between CD4 stages and μ_{ij} the mortality. The rate σ_{rev} represents reversion of NNRTI-resistance when no more drug pressure is exerted, while σ_{res}^{NNRTI} and σ_{res}^{NRTI} represents the rates of acquiring NNRTI resistance and NRTI resistance, respectively, when an individual is failing NNRTI-based treatment. To model new infections, we used β_u and β_d the respective monthly number of sexual contacts among undiagnosed and diagnosed individuals, $\rho_{k,k}$ the assumed proportion of heterosexual individuals within men and women and $\nu_{k,k'}$ the probability of HIV transmission per sexual act. Finally, we also used a function $\delta(x)$, which is given by :

$$\delta(x) = \begin{cases} -1 & \text{if } x = 0, \\ 1 & \text{if } x = 1. \end{cases} \tag{18}$$

$$\begin{aligned}
\dot{I}^{jklm}(t) = &- \nu_{CD4}^{I,j} \cdot I^{jklm}(t) \mathbb{1}_{j \leq 3} + \nu_{CD4}^{I,j-1} \cdot I^{(j-1)klm}(t) \mathbb{1}_{j \geq 2} \\
&+ \beta_u \left(\rho_{1-k,k} \nu_{1-k,k} \frac{Susc_k}{N_k} Inf_u^{(1-k)l} + \rho_{k,k} \nu_{k,k} \frac{Susc_k}{N_k} Inf_u^{kl} \right) \mathbb{1}_{j=1} \\
&+ \beta_d \left(\rho_{1-k,k} \nu_{1-k,k} \frac{Susc_k}{N_k} Inf_d^{(1-k)l} + \rho_{k,k} \nu_{k,k} \frac{Susc_k}{N_k} Inf_d^{kl} \right) \mathbb{1}_{j=1} \\
&- \gamma_{I \to D}^{jk}(t) \cdot I^{jklm}(t) - \delta(l) \cdot \sigma_{rev} \cdot I^{jk1m}(t) - \mu_I^j \cdot I^{jklm}(t),
\end{aligned}$$

$$\begin{aligned}
\dot{D}_{elig}^{jklm}(t) = &- \nu_{CD4}^{D,j} \cdot D_{elig}^{jklm}(t) \mathbb{1}_{j \leq 3} + \nu_{CD4}^{D,j} \cdot D_{elig}^{(j-1)klm}(t) \mathbb{1}_{j \geq 2} \\
&- (\gamma_{D \to T_1}^{j,elig}(t) + \gamma_{D \to T_2}^{jk}(t) + \gamma_{D \to T_3}^{jk}(t)) \cdot D_{elig}^{jklm}(t) \\
&+ p_k \gamma_{I \to D}^{jk}(t) \cdot I^{jklm}(t) - \delta(l) \cdot \sigma_{rev} \cdot D_{elig}^{jk1m}(t) - \mu_D^j \cdot D_{elig}^{jklm}(t),
\end{aligned}$$

$$\begin{aligned}
\dot{D}_{inel}^{jklm}(t) = &- \nu_{CD4}^{D,j} \cdot D_{inel}^{jklm}(t) \mathbb{1}_{j \leq 3} + \nu_{CD4}^{D,j} \cdot D_{inel}^{(j-1)klm}(t) \mathbb{1}_{j \geq 2} \\
&- \gamma_{D \to T_1}^{j,inel}(t) \cdot D_{inel}^{jklm}(t) \\
&+ (1 - p_k) \gamma_{I \to D}^{jk}(t) \cdot I^{jklm}(t) - \delta(l) \cdot \sigma_{rev} \cdot D_{inel}^{jk1m}(t) - \mu_D^j \cdot D_{inel}^{jklm}(t),
\end{aligned}$$

$$\begin{aligned}
\dot{T}_{1,elig}^{jklm}(t) = &\left(\nu_{CD4}^{T_1,j-1} \cdot T_{1,elig}^{(j-1)klm}(t) - \tilde{\nu}_{CD4}^{T_1,j-1} \cdot T_{1,elig}^{jklm}(t) \right) \mathbb{1}_{j \geq 2} \\
&+ \left(\tilde{\nu}_{CD4}^{T_1,j} \cdot T_{1,elig}^{(j+1)klm}(t) - \nu_{CD4}^{T_1,j} \cdot T_{1,elig}^{jklm}(t) \right) \mathbb{1}_{j \leq 3} \\
&- (\gamma_{T_1 \to S_1}^{jl} + \gamma_{T_1 \to F_1}^{jl}) \cdot T_{1,elig}^{jklm}(t) + \gamma_{D \to T_1}^{jk}(t) \cdot D_{elig}^{jklm}(t) - \mu_{T_1}^j \cdot T_{1,elig}^{jklm}(t)
\end{aligned}$$

$$\begin{aligned}
\dot{S}_{1,elig}^{jklm}(t) = &- \tilde{\nu}_{CD4}^{S_1,j-1} \cdot S_{1,elig}^{jklm}(t) \mathbb{1}_{j \geq 2} + \tilde{\nu}_{CD4}^{S_1,j} \cdot S_{1,elig}^{(j+1)klm}(t) \mathbb{1}_{j \leq 3} \\
&- \gamma_{S_1 \to F_1}^{jl} \cdot S_{1,elig}^{jklm}(t) + \gamma_{T_1 \to S_1}^{jl} \cdot T_{1,elig}^{jklm}(t) + \gamma_{F_1 \to S_1}^{jl} \cdot F_{1,elig}^{jklm}(t) - \mu_{S_1}^j \cdot S_{1,elig}^{jklm}(t) \\
&- \gamma_{S_1 \to S_3}(t) \cdot S_{1,elig}^{jklm}(t),
\end{aligned}$$

$$\begin{aligned}
\dot{F}_{1,elig}^{jklm}(t) = &\nu_{CD4}^{F_1,j-1} \cdot F_{1,elig}^{(j-1)klm}(t) \mathbb{1}_{j \geq 2} - \nu_{CD4}^{F_1,j} \cdot F_{1,elig}^{jklm}(t) \mathbb{1}_{j \leq 3} \\
&+ \delta(l) \cdot \sigma_{res}^{NNRTI} \cdot F_{1,elig}^{jk0m}(t) + \delta(m) \cdot \sigma_{res}^{NRTI} \cdot F_{1,elig}^{jkl0}(t) \\
&- (\gamma_{F_1 \to S_1}^{jl} + \gamma_{F_1 \to T_2}^{j,elig}(t)) \cdot F_{1,elig}^{jklm}(t) + \gamma_{S_1 \to F_1}^{jl} \cdot S_{1,elig}^{jklm}(t) + \gamma_{T_1 \to F_1}^{jl} \cdot T_{1,elig}^{jklm}(t) \\
&- \mu_{F_1}^j \cdot F_{1,elig}^{jklm}(t) - \gamma_{F_1 \to T_3}^j(t) \cdot F_{1,elig}^{jklm}(t),
\end{aligned}$$

$$\begin{aligned}
\dot{T}_{1,inel}^{jklm}(t) = &\left(\nu_{CD4}^{T_1,j-1} \cdot T_{1,inel}^{(j-1)klm}(t) - \tilde{\nu}_{CD4}^{T_1,j-1} \cdot T_{inel}^{jklm}(t) \right) \mathbb{1}_{j \geq 2} \\
&+ \left(\tilde{\nu}_{CD4}^{T_1,j} \cdot T_{1,inel}^{(j+1)klm}(t) - \nu_{CD4}^{T_1,j} \cdot T_{1,inel}^{jklm}(t) \right) \mathbb{1}_{j \leq 3} \\
&- (\gamma_{T_1 \to S_1}^{jl} + \gamma_{T_1 \to F_1}^{jl}) \cdot T_{1,inel}^{jklm}(t) + \gamma_{D \to T_1}^{jk}(t) \cdot D_{inel}^{jklm}(t) - \mu_{T_1}^j \cdot T_{1,inel}^{jklm}(t),
\end{aligned}$$

$$
\begin{aligned}
\dot{S}_{1,inel}^{jklm}(t) =& -\tilde{\nu}_{CD4}^{S_1,j-1} \cdot S_{1,inel}^{jklm}(t)\mathbb{1}_{j\geq 2} + \tilde{\nu}_{CD4}^{S_1,j} \cdot S_{1,inel}^{(j+1)klm}(t)\mathbb{1}_{j\leq 3} \\
& - \gamma_{S_1\to F_1}^{jl} \cdot S_{1,inel}^{jklm}(t) + \gamma_{T_1\to S_1}^{jl} \cdot T_{1,inel}^{jklm}(t) + \gamma_{F_1\to S_1}^{jl} \cdot F_{1,inel}^{jklm}(t) - \mu_{S_1}^{j} \cdot S_{1,inel}^{jklm}(t),
\end{aligned}
$$

$$
\begin{aligned}
\dot{F}_{1,inel}^{jklm}(t) =& \nu_{CD4}^{F_1,j-1} \cdot F_{1,inel}^{(j-1)klm}(t)\mathbb{1}_{j\geq 2} - \nu_{CD4}^{F_1,j} \cdot F_{1,inel}^{jklm}(t)\mathbb{1}_{j\leq 3} \\
& + \delta(l) \cdot \sigma_{res}^{NNRTI} \cdot F_{1,inel}^{jk0m}(t) + \delta(m) \cdot \sigma_{res}^{NRTI} \cdot F_{1,inel}^{jkl0}(t) \\
& - (\gamma_{F_1\to S_1}^{jl} + \gamma_{F_1\to T_2}^{j,inel}) \cdot F_{1,inel}^{jklm}(t) + \gamma_{S_1\to F_1}^{jl} \cdot S_{1,inel}^{jklm}(t) + \gamma_{T_1\to F_1}^{jl} \cdot T_{1,inel}^{jklm}(t) \\
& - \mu_{F_1}^{j} \cdot F_{1,inel}^{jklm}(t),
\end{aligned}
$$

$$
\begin{aligned}
\dot{T}_{2}^{jklm}(t) =& \left(\nu_{CD4}^{T_2,j-1} \cdot T_{2}^{(j-1)klm}(t) - \tilde{\nu}_{CD4}^{T_2,j-1} \cdot T_{2}^{jklm}(t)\right)\mathbb{1}_{j\geq 2} + \\
& \left(\tilde{\nu}_{CD4}^{T_2,j} \cdot T_{2}^{(j+1)klm}(t) - \nu_{CD4}^{T_2,j} \cdot T_{2}^{jklm}(t)\right)\mathbb{1}_{j\leq 3} \\
& - (\gamma_{T_2\to S_2}^{j} + \gamma_{T_2\to F_2}^{j}) \cdot T_{2}^{jklm}(t) + \gamma_{F_1\to T_2}^{j,elig}(t) \cdot F_{1,elig}^{jklm}(t) + \gamma_{F_1\to T_2}^{j,inel} \cdot F_{1,inel}^{jklm}(t) \\
& - \mu_{T_2}^{j} \cdot T_{2}^{jklm}(t),
\end{aligned}
$$

$$
\begin{aligned}
\dot{S}_{2}^{jklm}(t) =& -\tilde{\nu}_{CD4}^{S_2,j-1} \cdot S_{2}^{jklm}(t)\mathbb{1}_{j\geq 2} + \tilde{\nu}_{CD4}^{S_2,j} \cdot S_{2}^{(j+1)klm}(t)\mathbb{1}_{j\leq 3} \\
& - \gamma_{S_2\to F_2}^{j} \cdot S_{2}^{jklm}(t) + \gamma_{T_2\to S_2}^{j} \cdot T_{2}^{jklm}(t) + \gamma_{F_2\to S_2}^{j} \cdot F_{2}^{jklm}(t) - \mu_{S_2}^{j} \cdot S_{2}^{jklm}(t),
\end{aligned}
$$

$$
\begin{aligned}
\dot{F}_{2}^{jklm}(t) =& \nu_{CD4}^{F_2,j-1} \cdot F_{2}^{(j-1)klm}(t)\mathbb{1}_{j\geq 2} - \nu_{CD4}^{F_2,j} \cdot F_{2}^{jklm}(t)\mathbb{1}_{j\leq 3} \\
& - \gamma_{F_2\to S_2}^{j} \cdot F_{2}^{jklm}(t) + \gamma_{S_2\to F_2}^{j} \cdot S_{2}^{jklm}(t) + \gamma_{T_2\to F_2}^{j} \cdot T_{2}^{jklm}(t) - \mu_{F_2}^{j} \cdot F_{2}^{jklm}(t),
\end{aligned}
$$

$$
\begin{aligned}
\dot{T}_{3}^{jklm}(t) =& \left(\nu_{CD4}^{T_1,j-1} \cdot T_{3}^{(j-1)klm}(t) - \tilde{\nu}_{CD4}^{T_1,j-1} \cdot T_{3}^{jklm}(t)\right)\mathbb{1}_{j\geq 2} \\
& + \left(\tilde{\nu}_{CD4}^{T_1,j} \cdot T_{3}^{(j+1)klm}(t) - \nu_{CD4}^{T_1,j} \cdot T_{3}^{jklm}(t)\right)\mathbb{1}_{j\leq 3} \\
& - (\gamma_{T_3\to S_3}^{j0} + \gamma_{T_3\to F_3}^{j0}) \cdot T_{3}^{jklm}(t) + \gamma_{D\to T_3}^{jk}(t) \cdot D_{elig}^{jklm}(t) - \mu_{T_1}^{j} \cdot T_{3}^{jklm}(t) \\
& + \gamma_{F_1\to T_3}^{j}(t) \cdot F_{1,elig}^{jklm}(t),
\end{aligned}
$$

$$
\begin{aligned}
\dot{S}_{3}^{jklm}(t) =& -\tilde{\nu}_{CD4}^{S_1,j-1} \cdot S_{3}^{jklm}(t)\mathbb{1}_{j\geq 2} + \tilde{\nu}_{CD4}^{S_1,j} \cdot S_{3}^{(j+1)klm}(t)\mathbb{1}_{j\leq 3} \\
& - \gamma_{S_3\to F_3}^{j0} \cdot S_{3}^{jklm}(t) + \gamma_{T_3\to S_3}^{j0} \cdot T_{3}^{jklm}(t) + \gamma_{F_3\to S_3}^{j0} \cdot F_{3}^{jklm}(t) - \mu_{S_1}^{j} \cdot S_{3}^{jklm}(t) \\
& + \gamma_{S_1\to S_3}(t) \cdot S_{1,elig}^{jklm}(t),
\end{aligned}
$$

$$
\begin{aligned}
\dot{F}_{3}^{jklm}(t) =& \nu_{CD4}^{F_1,j-1} \cdot F_{3}^{(j-1)klm}(t)\mathbb{1}_{j\geq 2} - \nu_{CD4}^{F_1,j} \cdot F_{3}^{jklm}(t)\mathbb{1}_{j\leq 3} \\
& - (\gamma_{F_3\to S_3}^{jm} + \gamma_{F_1\to T_2}^{j}) \cdot F_{3}^{jklm}(t) + \gamma_{S_3\to F_3}^{jm} \cdot S_{3}^{jklm}(t) + \gamma_{T_3\to F_3}^{jm} \cdot T_{3}^{jklm}(t) \\
& - \mu_{F_1}^{j} \cdot F_{3}^{jklm}(t). \qquad (19)
\end{aligned}
$$

5 Sensitivity analysis and additional results

The main sensitivity analysis reflects the uncertainty about eight parameters related to resistance and HIV-transmission. The 95% sensitivity ranges in 2040 for each scenario are shown in Fig 3 of the main manuscript. The evolution of uncertainty over time is represented in Fig C, which displays the 95% sensitivity ranges from 2005 to 2040 for each scenario. The difference in NNRTI TDR levels over time between the different scenarios of DTG-introduction and the scenario where DTG is not introduced is displayed in Fig D with the 95% sensitivity ranges. Fig E diplays the predicted percentage of women failing NNRTI-based ART after 1 and 2 years of ART in 2025 and 2035, depending on the scenario of the rollout of DTG-based ART. Finally, we simulated three sensitivity analyses in order to investigate 1) the impact of the Treat-All policy, 2) the impact of treatment interruption, and 3) the impact of NRTI-resistance and higher efficacy of DTG.

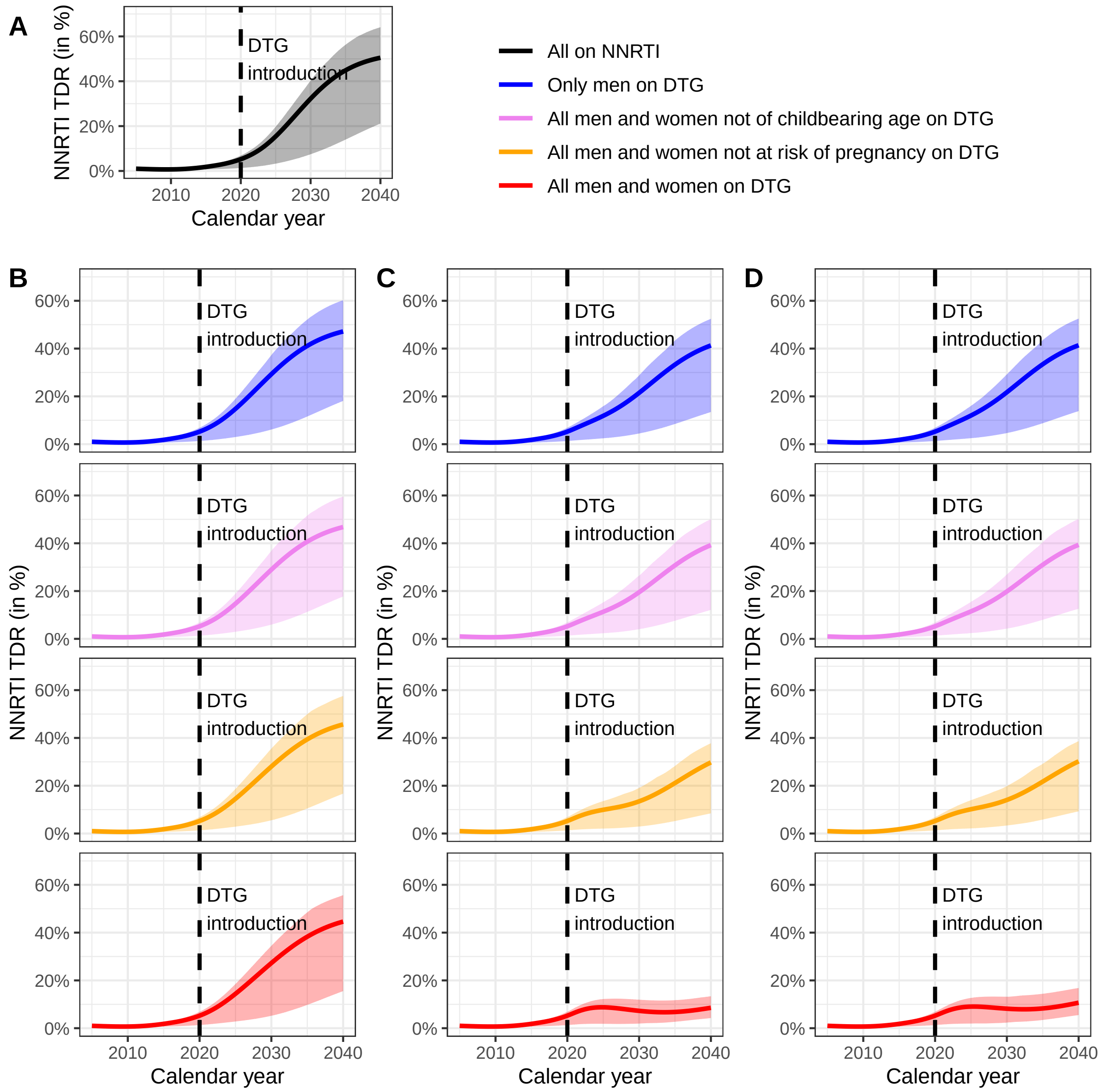

Fig C: Level of NNRTI PDR according to different levels of DTG-eligible women (colors), and different strategies of DTG-introduction. Panel A: no DTG-introduction; panel B: DTG used as a first-line regimen; panel C: DTG used for all patients; panel D: DTG used for all patients, assuming an OR of failure of 2 when having NRTI-resistance. The solid lines correspond to the simulations with the fixed parameter values and the shaded areas represent the 95% sensitivity ranges.

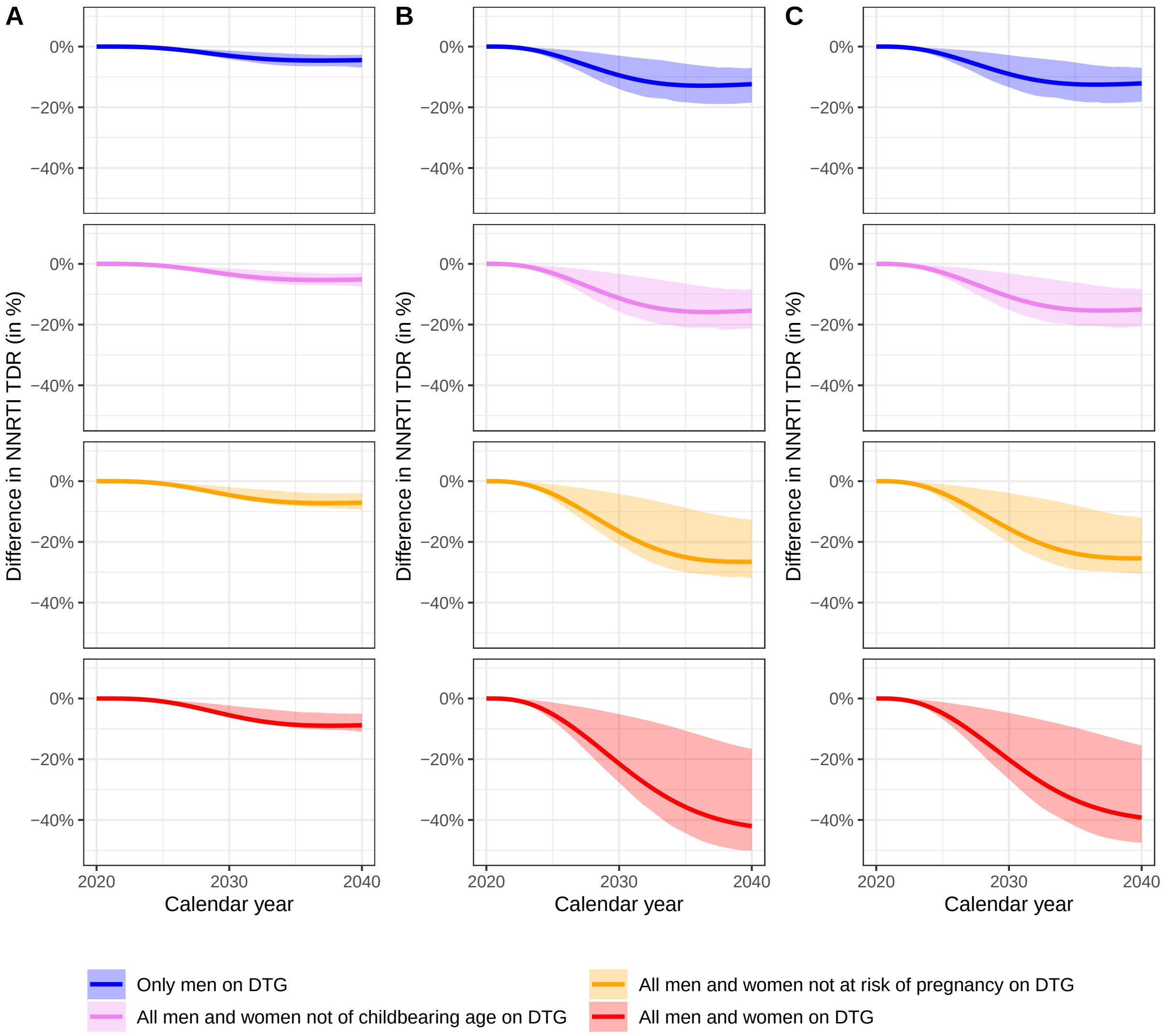

Fig D: Difference in level of NNRTI PDR from 2020 to 2040 between the different strategies of DTG-introduction and the scenario where DTG is not introduced. Panel A: DTG used as a first-line regimen; panel B: DTG used for all patients; panel C: DTG used for all patients, assuming an OR of DTG-failure of 2 when having NRTI-resistance. The solid lines correspond to the simulations with the fixed parameter values and the shaded areas represent the 95% sensitivity ranges.

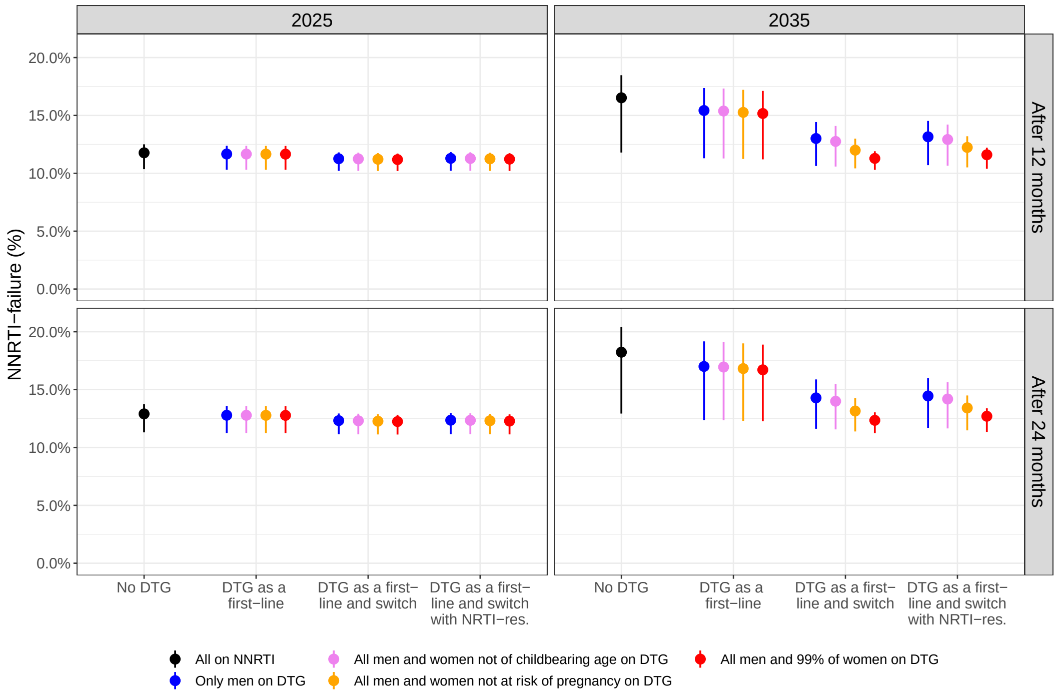

Fig E: Predicted percentage of women failing NNRTI-based ART after 1 and 2 years of ART in 2025 and 2035, depending on the scenario of the rollout of DTG based-ART.

5.1 Effect of no Treat-All policy

We previously assumed that the Treat-All policy increased the treatment initiation rates for people with $CD4 > 200$ cells/μL from 2017 to 2022. Here, we investigated the scenario where the Treat-All policy does not have any impact on the treatment initiation rates (which is equivalent to assuming no Treat-All policy, see FigF). Globally, assuming a Treat-All policy increases the levels of NNRTI PDR for each scenario, but does not change our conclusion (see FigG).

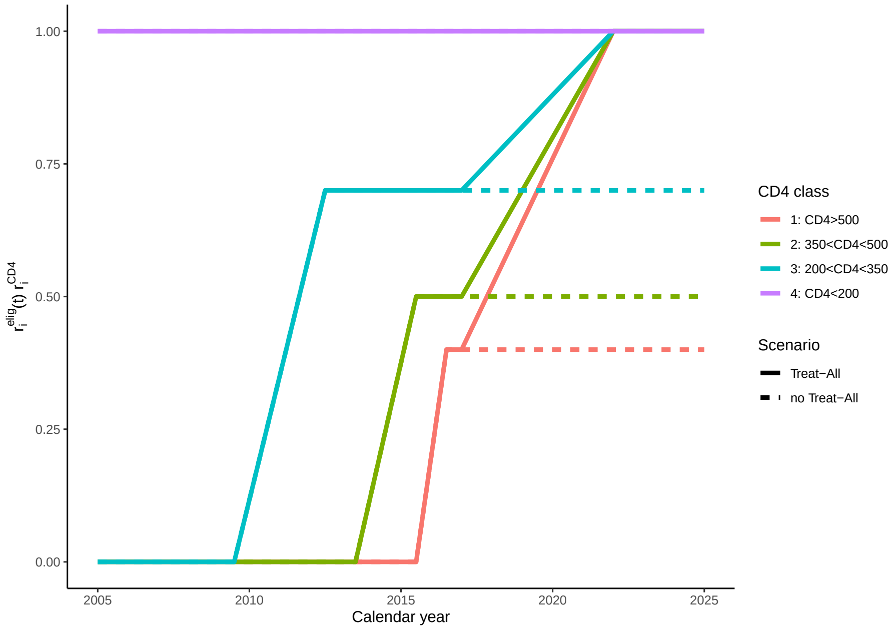

Fig F: $r_i^{elig}(t) \cdot r_i^{CD4}$ represents the level of treatment eligibility $r_i^{elig}(t)$ multiplied by r_i^{CD4}, representing the decrease in treatment initiation rate in CD4 class i relative to the fourth CD4 class ($CD4 < 200$ cells/μL). In the scenario where we assumed no impact of the Treat-All policy on the treatment initiation rates, the rates remain unchanged from 2016.

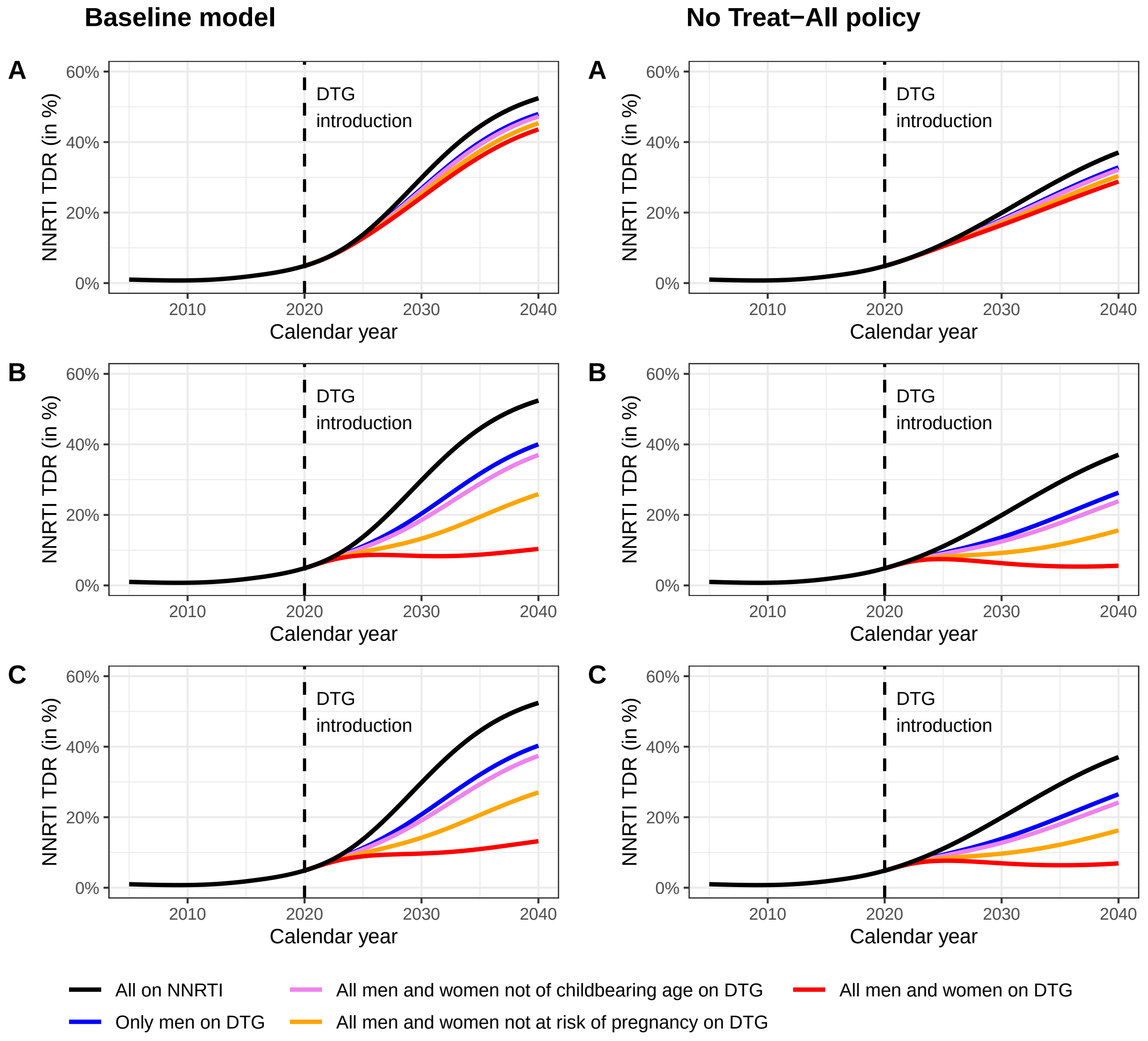

Fig G: Levels of NNRTI resistance when assuming increase in treatment initiation rates due to the Treat-All policy ("Baseline Model") and when assuming identical treatment initiation rates from 2017 ("No Treat-All policy"). Dolutegravir is introduced in 2020 under three scenarios: DTG as first-line regimen for ART-initiators (panel A), DTG for all patients (panel B), DTG for all patients, assuming an OR of failure of 2 when having NRTI-resistance (panel C), and with different eligibility criteria for women (colors).

5.2 Effect of treatment interruption

We introduced treatment interruption rates for the three ART regimens. Table G shows these rates estimated from IeDEA-SA data [1]. The introduction of treatment interruption did not substantially change the results (see Fig H).

Table G: Treatment interruption rates. Rates are in month^{-1}

Parameter	Description	CD4 class			
		1	2	3	4
$1/\gamma_{T\to D}$	Time from $T_1/T_2/T_3$ to D	414	322	172	156
$1/\gamma_{S\to D}$	Time from $S_1/S_2/S_3$ to D	2069	1241	759	368
$1/\gamma_{F\to D}$	Time from $F_1/F_2/F_3$ to D	621	478	285	129

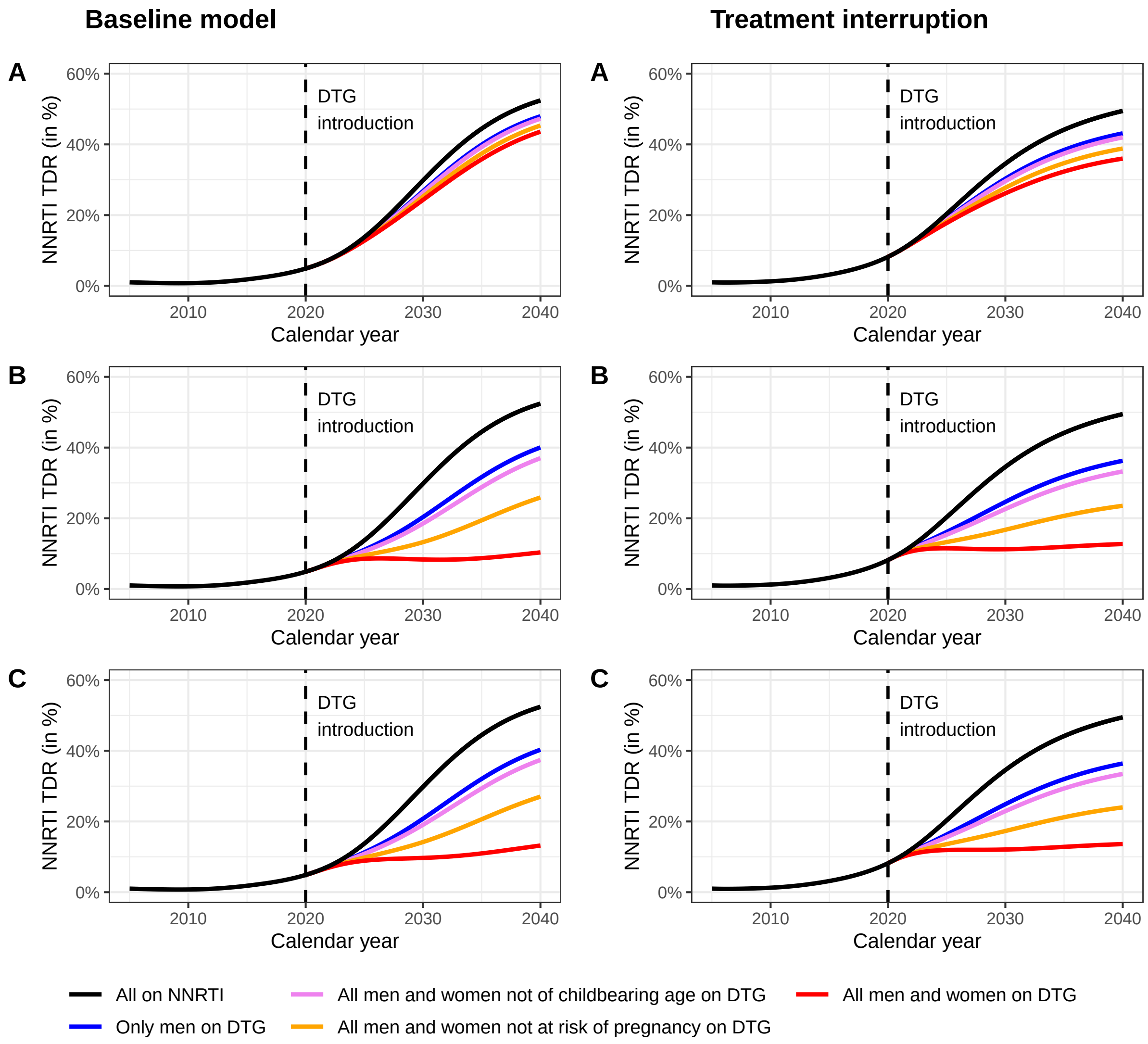

Fig H: Levels of NNRTI resistance using the baseline model ("Baseline Model") and when including treatment interruption ("Treatment interruption"). Dolutegravir is introduced in 2020 under three scenarios: DTG as first-line regimen for ART-initiators (panel A) or DTG for all patients (panel B), DTG for all patients, assuming an OR of failure of 2 when having NRTI-resistance (panel C), and with different eligibility criteria for women (colors).

5.3 Effect of NRTI-resistance and higher efficacy of DTG

We assessed the impact of NRTI resistance and higher efficacy of DTG-based regimen on the level of NNRTI PDR. We investigated three different scenarios regarding the impact of NRTI-resistance: 1) no impact (i.e OR of failure between NRTI-resistant and NRTI-susceptible individuals equals 1), 2) OR=2, and 3) OR=5. A meta-analysis comparing DTG-monotherapy with DTG-dual therapy found an odds ratio of failure of 13.9 after 48 weeks (8.9% vs 0.7% of failure, respectively) [17] However, we expect lower difference between NRTI-resistant and -susceptible individuals, as some activity of the NRTI-backbones are observed even in resistant individuals [15]. Another study comparing DTG-efficacy according to the presence of specific NRTI-mutations found a HR of 3.23 (95%CI: 0.27-38.40) when having the K65R mutation and a HR of 0.99 (95%CI: 0.19-5.21) when having the M184V, suggesting low impact of NRTI-resistance on DTG-failure [16].
We also investigated three different scenarios regarding the efficacy of DTG compared with NNRTI: 1) OR of failure between NNRTI- and DTG-based regimen of 1.02, 2) OR=2, and 3) OR=5. The first scenario refers to the results of the NAMSAL study after the adjusting for CD4 counts (see Section 2.5). The two other scenarios were investigated in view of the higher efficacy of DTG compared with NNRTI found in some studies [26].

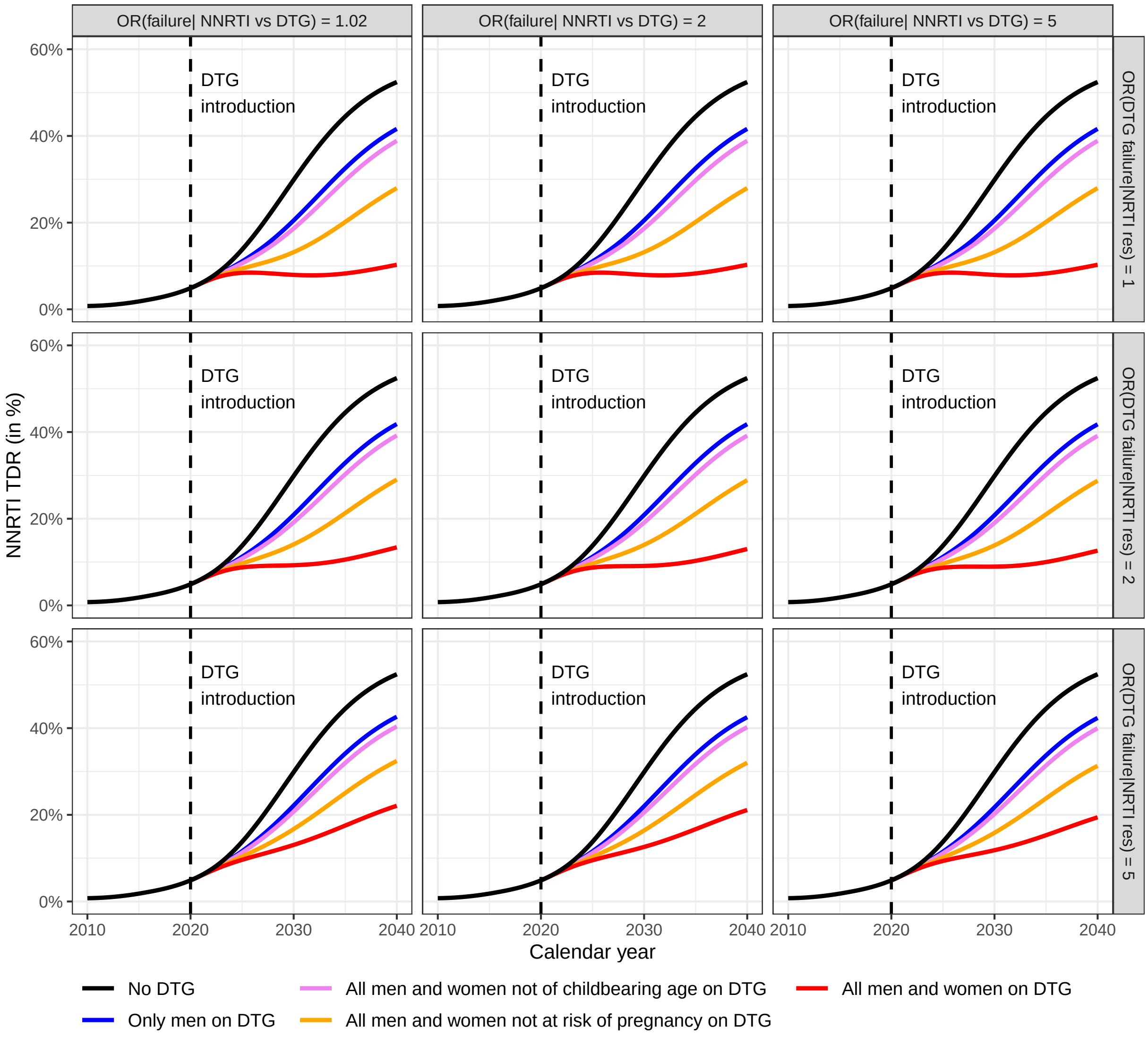

Fig I: Levels of NNRTI TDR from 2010 to 2040, when assuming that DTG is used both as first-line and switch regimens. Different impacts of NRTI-resistance on DTG-failure (horizontally) and different DTG-efficacies (vertically) are investigated.

References

[1] Egger M, Ekouevi DK, Williams C, Lyamuya RE, Mukumbi H, Braitstein P, et al. Cohort Profile: The international epidemiological databases to evaluate AIDS (IeDEA) in sub-Saharan Africa. International Journal of Epidemiology. 2012 oct;41(5):1256–1264. Available from: http://www.ncbi.nlm.nih.gov/pubmed/21593078http://www.pubmedcentral.nih.gov/articlerender.fcgi?artid=PMC3465765https://academic.oup.com/ije/article-lookup/doi/10.1093/ije/dyr080.

[2] Johnson LF, Dorrington RE. Thembisa version 4.1: A model for evaluating the impact of HIV/AIDS in South Africa; 2018. Available from: https://www.thembisa.org/publications.

[3] Mangal TD, UNAIDS Working Group on CD4 Progression and Mortality Amongst HIV Seroconverters including the CASCADE Collaboration in EuroCoord. Joint estimation of CD4+ cell progression and survival in untreated individuals with HIV-1 infection. AIDS. 2017 may;31(8):1073–1082. Available from: http://www.ncbi.nlm.nih.gov/pubmed/28301424http://www.pubmedcentral.nih.gov/articlerender.fcgi?artid=PMC5414573http://insights.ovid.com/crossref?an=00002030-201705150-00003.

[4] Hauser A, Kusejko K, Johnson LF, Wandeler G, Riou J, Goldstein F, et al. Bridging the gap between HIV epidemiology and antiretroviral resistance evolution: Modelling the spread of resistance in South Africa. PLOS Computational Biology. 2019 jun;15(6):e1007083. Available from: http://dx.plos.org/10.1371/journal.pcbi.1007083.

[5] Moorhouse M, Maartens G, Venter WDF, Moosa MY, Steegen K, Jamaloodien K, et al. Third-Line Antiretroviral Therapy Program in the South African Public Sector: Cohort Description and Virological Outcomes. Journal of acquired immune deficiency syndromes (1999). 2019 jan;80(1):73–78. Available from: http://www.ncbi.nlm.nih.gov/pubmed/30334876http://www.pubmedcentral.nih.gov/articlerender.fcgi?artid=PMC6319697.

[6] Orrell C, Walensky RP, Losina E, Pitt J, Freedberg KA, Wood R. HIV type-1 clade C resistance genotypes in treatment-naive patients and after first virological failure in a large community antiretroviral therapy programme. Antiviral therapy. 2009;14(4):523–31. Available from: http://www.ncbi.nlm.nih.gov/pubmed/19578237http://www.pubmedcentral.nih.gov/articlerender.fcgi?artid=PMC3211093.

[7] Sigaloff KCE, Ramatsebe T, Viana R, de Wit TFR, Wallis CL, Stevens WS. Accumulation of HIV Drug Resistance Mutations in Patients Failing First-Line Antiretroviral Treatment in South Africa. AIDS Research and Human Retroviruses. 2012 feb;28(2):171–175. Available from: http://www.liebertpub.com/doi/10.1089/aid.2011.0136.

[8] Wallis CL, Mellors JW, Venter WDF, Sanne I, Stevens W. Varied Patterns of HIV-1 Drug Resistance on Failing First-Line Antiretroviral Therapy in South Africa. JAIDS Journal of Acquired Immune Deficiency Syndromes. 2010 apr;53(4):480–484. Available from: https://insights.ovid.com/crossref?an=00126334-201004010-00007.

[9] Manasa J, Lessells RJ, Skingsley A, Naidu KK, Newell ML, McGrath N, et al. High-Levels of Acquired Drug Resistance in Adult Patients Failing First-Line Antiretroviral Therapy in a Rural HIV Treatment Programme in KwaZulu-Natal, South Africa. PLoS ONE. 2013 aug;8(8):e72152. Available from: https://dx.plos.org/10.1371/journal.pone.0072152.

[10] van Zyl GU, van der Merwe L, Claassen M, Zeier M, Preiser W. Antiretroviral resistance patterns and factors associated with resistance in adult patients failing NNRTI-based regimens in the western cape, South Africa. Journal of Medical Virology. 2011 oct;83(10):1764–1769. Available from: http://doi.wiley.com/10.1002/jmv.22189.

[11] Hauser A. Acquired HIV drug resistance mutations on first-line antiretroviral therapy in Southern Africa: Bayesian evidence synthesis;. Available from: https://github.com/anthonyhauser/ADR-meta-analysis.

[12] Yang WL, Kouyos RD, Böni J, Yerly S, Klimkait T, Aubert V, et al. Persistence of Transmitted HIV-1 Drug Resistance Mutations Associated with Fitness Costs and Viral Genetic Backgrounds. PLOS Pathogens. 2015 mar;11(3):e1004722. Available from: http://dx.plos.org/10.1371/journal.ppat.1004722.

[13] Wittkop L, Günthard HF, de Wolf F, Dunn D, Cozzi-Lepri A, de Luca A, et al. Effect of transmitted drug resistance on virological and immunological response to initial combination antiretroviral therapy for HIV (EuroCoord-CHAIN joint project): a European multicohort study. The Lancet Infectious Diseases. 2011 may;11(5):363–371. Available from: http://www.ncbi.nlm.nih.gov/pubmed/21354861http://linkinghub.elsevier.com/retrieve/pii/S1473309911700329.

[14] Kuritzkes DR, Lalama CM, Ribaudo HJ, Marcial M, Meyer III WA, Shikuma C, et al. Preexisting Resistance to Nonnucleoside Reverse-Transcriptase Inhibitors Predicts Virologic Failure of an Efavirenz-Based Regimen in Treatment-Naive HIV-1–Infected Subjects. The Journal of Infectious Diseases. 2008 mar;197(6):867–870. Available from: https://academic.oup.com/jid/article-lookup/doi/10.1086/528802.

[15] Hakim JG, Thompson J, Kityo C, Hoppe A, Kambugu A, van Oosterhout JJ, et al. Lopinavir plus nucleoside reverse-transcriptase inhibitors, lopinavir plus raltegravir, or lopinavir monotherapy for second-line treatment of HIV (EARNEST): 144-week follow-up results from a randomised controlled trial. The Lancet Infectious Diseases. 2018 jan;18(1):47–57. Available from: http://www.thelancet.com/article/S1473309917306308/fulltexthttp://www.thelancet.com/article/S1473309917306308/abstracthttps://www.thelancet.com/journals/laninf/article/PIIS1473-3099(17)30630-8/abstract.

[16] Giacomelli A, Lai A, Franzetti M, Maggiolo F, Di Giambenedetto S, Borghi V, et al. No impact of previous NRTIs resistance in HIV positive patients switched to DTG+2NRTIs under virological control: Time of viral suppression makes the difference. Antiviral Research. 2019 dec;172. Available from: https://pubmed.ncbi.nlm.nih.gov/31629714/.

[17] Wandeler G, Buzzi M, Anderegg N, Sculier D, Béguelin C, Egger M, et al. Open Peer Review Virologic failure and HIV drug resistance on simplified, dolutegravir-based maintenance therapy: Systematic review and meta-analysis [version 2; peer review: 3 approved]. F1000 Research. 2019. Available from: https://doi.org/10.12688/f1000research.15995.1.

[18] Group TNAS. Dolutegravir-Based or Low-Dose Efavirenz–Based Regimen for the Treatment of HIV-1. New England Journal of Medicine. 2019 aug;381(9):816–826. Available from: http://www.nejm.org/doi/10.1056/NEJMoa1904340.

[19] Patel P, Borkowf CB, Brooks JT, Lasry A, Lansky A, Mermin J. Estimating per-act HIV transmission risk. AIDS. 2014 jun;28(10):1509–1519. Available from: http://www.ncbi.nlm.nih.gov/pubmed/24809629http://content.wkhealth.com/linkback/openurl?sid=WKPTLP:landingpage{&}an=00002030-201406190-00014.

[20] Anova Health Institute. Rapid Assessment of HIV Prevention, Care and Treatment Programming for MSM in South Africa; 2013.

[21] Maduna PH, Dolan M, Kondlo L, Mabuza H, Dlamini JN, Polis M, et al. Morbidity and Mortality According to Latest CD4+ Cell Count among HIV Positive Individuals in South Africa Who Enrolled in Project Phidisa. PLOS ONE. 2015 apr;10(4):e0121843. Available from: http://www.ncbi.nlm.nih.gov/pubmed/25856495http://www.pubmedcentral.nih.gov/articlerender.fcgi?artid=PMC4391777http://dx.plos.org/10.1371/journal.pone.0121843.

[22] Brennan AT, Maskew M, Sanne I, Fox MP. The interplay between CD4 cell count, viral load suppression and duration of antiretroviral therapy on mortality in a resource-limited setting. Tropical medicine & international health : TM & IH. 2013 may;18(5):619–31. Available from: http://www.ncbi.nlm.nih.gov/pubmed/23419157http://www.pubmedcentral.nih.gov/articlerender.fcgi?artid=PMC3625450.

[23] Kühnert D, Kouyos R, Shirreff G, Pečerska J, Scherrer AU, Böni J, et al. Quantifying the fitness cost of HIV-1 drug resistance mutations through phylodynamics. PLOS Pathogens. 2018 feb;14(2):e1006895. Available from: http://dx.plos.org/10.1371/journal.ppat.1006895.

[24] Rhee SY, Varghese V, Holmes SP, Van Zyl GU, Steegen K, Boyd MA, et al. Mutational Correlates of Virological Failure in Individuals Receiving a WHO-Recommended Tenofovir-Containing First-Line Regimen: An International Collaboration. EBioMedicine. 2017 apr;18:225–235. Available from: https://pubmed.ncbi.nlm.nih.gov/28365230/.

[25] World Bank. Contraceptive prevalence, any methods (% of women ages 15-49) | Data; 2015. Available from: https://data.worldbank.org/indicator/SP.DYN.CONU.ZS?locations=ZA.

[26] Snedecor SJ, Radford M, Kratochvil D, Grove R, Punekar YS. Comparative efficacy and safety of dolutegravir relative to common core agents in treatment-naïve patients infected with HIV-1: A systematic review and network meta-analysis. BMC Infectious Diseases. 2019 may;19(1). Available from: /pmc/articles/PMC6543679/?report=abstracthttps://www.ncbi.nlm.nih.gov/pmc/articles/PMC6543679/.
