# Supplementary material for: Impact of scaling up dolutegravir on antiretroviral resistance in South Africa: A modeling study
Source: PLoS Med. 2020 Dec 14;17(12):e1003397. doi: 10.1371/journal.pmed.1003397 (PMC7735592; doi:10.1371/journal.pmed.1003397)
Supplement: Attachment — Submitted filename: Response_to_reviewers Hauser.pdf [file pmed.1003397.s002.pdf]

Authors' response to referee comments: Impact of Scaling up Dolutegravir on Antiretroviral Resistance in South Africa: A Mathematical Modelling Study

Hauser et al.

Requests from the editors:

***Trivial request, but journal structure for abstract subsections should be Background, Methods and Findings, Conclusions (Methods and Findings a single subsection).**

This has been modified.

*** In the last sentence of the Abstract Methods and Findings section, we'd suggest including a brief description of any key limitation(s) of the study methods/findings.**

We include the following sentences at the end of the Methods and Findings section :
"Limitations include high uncertainty due to the long-term predictions and the current scarcity of knowledge about DTG efficacy in South Africa."

***At this stage, we ask that you include a short, non-technical Author Summary of your research to make findings accessible to a wide audience that includes both scientists and non-scientists. The Author Summary should immediately follow the Abstract in your revised manuscript. This text is subject to editorial change and should be distinct from the scientific abstract. Please see our author guidelines for more information: https://journals.plos.org/plosmedicine/s/revising-your-manuscript#loc-author-summary**

Thank you. Done.

Comments from the reviewers:

Reviewer #1
A modelling study to predict the impact of various scenarios of DTG-based ART rollout in South Africa on the levels of pretreatment NNRTI-resistance. The model uses the established MARISA model based on 5 HIV cohorts. The work is well performed with many relevant scenarios being modelled, suggesting that DTG rollout for all will lead to a substantial drop in NNRTI-resistance.

Major comment: L239-259: The authors here describe as limitations what I find a major omission of the model, or a missed opportunity. The impact of co-existing NRTI-resistance on DTG resistance development, (long-term) DTG effectiveness in real-life programmatic conditions, the influence of non-B subtypes on DTG resistance patterns, the lack of stringent VL monitoring allowing for ongoing VF, the resultant potential of transmission of DTG-resistant trains, who in turn can compromise first-line DTG-based regimens. In my view, the current model has taken a fairly narrow approach focusing on NNRTI resistance as the main outcome; it would be more useful and timely to undertake a serious attempt (even given limited available data) to include the broader outcomes connected to DTG effectiveness and resistance.
We thank the reviewer for pointing out these important issues. We had not included NRTI-resistance in the previous version of the MARISA model for two reasons. First, because the model, which takes into account the different treatment options, CD4 counts, gender and NNRTI-resistance, was already quite complex and difficult to parametrize, and little is known about the specific impact of NRTI-resistance mutations on DTG-treatment in real-world settings. Second, because NRTI-resistance only

has an indirect effect on levels of NNRTI-resistance, by increasing risk of DTG-failure, which help the propagation of NNRTI-resistance, as individuals having NRTI-resistance often also have NNRTI-resistance. Following the referee's suggestion, we included, in the new version of the model, additional scenarios where we model acquired NRTI-resistance and assumed an impact of NRTI-resistance on DTG-efficacy. For more details, see last paragraph of "Calibration and extension of the MARISA model" section in "Methods", where we wrote:

"In the adapted MARISA model, we also added a fifth NRTI-resistance dimension to model the impact of NRTI-resistance on DTG-efficacy. NRTI-resistance is defined as having resistance to both tenofovir (TDF) and lamivudine/emtricitabine (3TC/FTC), the two backbones that are usually combined with DTG (see S1 Text, Section 2.5). We assume that NRTI-resistance is acquired when failing NNRTI-based regimen. In view of the low levels of NRTI PDR that are observed in Africa and its rapid reversion to wild-type, we assumed that it cannot be transmitted. We investigated different impacts of NRTI-resistance on DTG-based regimen and different DTG-efficacies. For this aim, we recalibrated the model so that it reflects different odds ratios (ORs) of DTG-failure between NRTI-susceptible and NRTI-resistant individuals (OR=1, OR=2, OR=3). In the main analysis, we assumed that DTG-efficacy was similar to the one observed in the NAMSAL study, corresponding to an OR of failure between NNRTI and DTG of 1.02, after adjusting for the different baseline characteristics of the two groups (see S1 Text, Section 2.5). Other DTG efficacy corresponding to an OR of 2 and 5 were investigated in a supplementary analysis (see S1 Text, Section 5.3). All code and manuscript are available from https://github.com/anthonyhauser/MARISA2."

Moreover, we assumed an odds ratio (OR) of DTG-failure of 2 between NRTI-resistant and NRTI-susceptible individuals (different ORs were investigated in S1 Text Section 5.3). Results were similar to the scenario where we assume no impact of NRTI-resistance (Fig 3B vs Fig 3C), with slightly higher levels of NNRTI-resistance.

We did not include DTG-resistance into the new version of the MARISA model, because little is known on the rate of acquisition of the different DTG-resistance mutations and even less on their impact on the efficacy of DTG. The few studies investigating these rates usually had a minimal number of people failing the DTG-based regimen. The most commonly observed DTG-resistance mutations are R263K and G118R, but these mutations alone confer little or moderate resistance to DTG. Some in-vitro studies suggest that when several mutations were combined, e.g. the Q148 mutations with G140 and/or E138 mutations, higher levels of resistance could be achieved, but the applicability of such findings in the in-vitro settings are still not clear [1].

Even if we do not explicitly model DTG-resistance, we explored different decreases in DTG-efficacy, which could either reflect the emergence of DTG-resistance or the lower efficacy in real-life programmatic conditions compared to RCTs in sensitivity analyses. This was done in Section 5.3 of S1 Text, where we assumed different ORs for DTG-failure (OR 1, 2 and 5), together with different ORs of ART-failure comparing NNRTI and DTG (OR of 1.02 as in NAMSAL, 2 and 5). Again, results were consistent with what we found when assuming no impact of NRTI-resistance, but nevertheless, they show that NRTI-resistance might increase NNRTI TDR.

The reviewer also mentioned the importance of modelling the impact of "the lack of stringent VL monitoring allowing for ongoing VF" on NNRTI-resistance. In Fig 4, we varied the switching rate from NNRTI to DTG and investigated its impact on NNRTI TDR. For DTG, we assumed a similar switching rate at failure as for NNRTI-regimen, which has been estimated with patients' information from IeDEA-SA collaboration. The very low switching rates (see S1 Text Table 2) reflects both the lack of stringent VL monitoring, as mentioned by the reviewer, and the limited access to second-line ART.

Minor comments:

Adding a scenario of transitioning ALL patients (1st and 2nd line) to DTG would be useful.
We did not include the scenario where people failing 2nd-line PI-based regimen switch to DTG for the following reasons. First, as very few individuals are on second-line ART (estimated at 4% in 2016 [2]), this would have little impact on the results. Second, as we already model the switching from failing DTG-based regimen to PI-based regimen, including the transitioning of patients from PI to DTG would allow patients currently on PI after a DTG-failure to switch back to DTG, which is not realistic.

Line 150: "Specifically, under the scenario of continued NNRTI-based ART as standard first-line therapy, NNRTI resistance would increase to 46.8% (95% sensitivity range: 19.7%-54.5%) by 2030 and 58.5% (32.5%-68.9%) by 2040 (Fig 3A). In my view, it would be unrealistic to assume that these high levels of NNRTI resistance would be accepted without any policy actions, either in terms of genotypic resistance testing, enhanced VL monitoring, or accelerated access to DTG. So the scenario modelled is too simplistic.
We completely agree with the reviewer that this scenario is very unlikely. We did not include it to predict the impact of a possible policy (as South Africa already started introducing DTG) but rather to model the natural evolution over time of NNRTI-resistance when there is no alternative to NNRTI-based first-line regimen. It identifies the highest level that NNRTI-resistance can reach, and allows us to assess the ability of various strategies of introducing DTG to decrease it. As an example, by comparing the black with the blue lines in Fig 3A, we observe the limited impact of only switching men to DTG, as both black and blue curves reach high levels of NNRTI-resistance in 2030-2040.

L156: "would stabilize NNRTI resistance at a low level, with a prevalence of 14.3% (3.5%-17.5%) by 2030 and 14.8% (6.6%-19.5%) by 2040 (Fig 3B)."
Actually, these levels of NNRTI resistance are not so low at all, and would still warrant a guideline change to standard non-NNRTI first-line according to current WHO guidelines.
We agree with the reviewer that the terminology regarding levels of resistance was not well chosen. We used "low" because these levels of resistance are low compared to the other scenarios. We now changed the terminology to meet WHO's definition: <5%: low, 5%-15%: moderate, >15% high. Of note, the updated analysis found slightly lower levels of NNRTI-resistance.

Line 21: " at risk of pregnancy" epidemiologist jargon, better say "of childbearing potential"
We use the terminology "women at risk of pregnancy" to characterize women of childbearing age not using contraception. As the terminology "women of childbearing potential" includes women of childbearing age who use contraception, we decided to keep the term "at risk of pregnancy". However, we added the following sentence to clarify the difference between "of childbearing age" (scenario b, in Section "Scenarios") and "at risk of pregnancy" (scenario c):
"Throughout the rest of the paper, scenarios a) and b) will be referred as "men and women of childbearing age" and "men and women at risk of pregnancy", respectively."

Reviewer #2:
Impact of Scaling up Dolutegravir on Antiretroviral Resistance in South Africa: A Mathematical Modelling Study

The manuscript by Hauser et al., describes how the scale-up of dolutegravir (DTG) will impact HIV drug resistance, with specific interest on non-nucleoside reverse transcriptase inhibitors (NNRTIs). Although modelling studies have their limitations, they are useful in predicting possible outcomes based on current knowledge. Hauser et al., performed a comprehensive analysis based on the MARISA model and highlighted some very important results, especially with regards to use of DTG in women of childbearing potential.

General Comments:

1. This paper clearly highlights that increasing access to DTG for women has a great impact on curbing NNRTI resistance, even when compared to increasing the rate of switching ART.

2. However, and as mentioned at the end of the discussion section, it would be useful in future analysis to extend the models resistance dimension to NRTI resistance, as NRTIs are the main drug class administered together with DTG. Compromised NRTI drugs (such as tenofovir) will pose a risk of DTG functional monotherapy.

We acknowledge that NRTI-resistance at switch might undermine DTG-efficacy, and thus play a role in the transmission of NNRTI-resistance. In the revised manuscript (Fig 3B/C and S1 Text Section 5.3), we added a scenario investigating different impacts of NRTI-resistance on DTG (OR=1 and OR=2, see Fig 3B/C). In a sensitivity analysis, we also varied the DTG-efficacy over a broader range (OR of failure between NNRTI and DTG going from 1 to 5) and assessed the combined effect of higher DTG-efficacy with different impacts of NRTI-resistance on DTG-based regimen. Overall, we find that assuming higher DTG efficacy does not affect the estimated level of NNRTI PDR, while assuming an impact of NRTI-resistance on DTG-efficacy will slightly increase the levels of NNRTI PDR estimated by the model. For more details, see response to major comment of reviewer 1 (above) and last paragraph of "Calibration and extension of the MARISA model" section in "Methods", where we wrote:

"In the adapted MARISA model, we also added a fifth NRTI-resistance dimension to model the impact of NRTI-resistance on DTG-efficacy. NRTI-resistance is defined as having resistance to both tenofovir (TDF) and lamivudine/emtricitabine (3TC/FTC), the two backbones that are usually combined with DTG (see S1 Text, Section 2.5). We assume that NRTI-resistance is acquired when failing NNRTI-based regimen. In view of the low levels of NRTI PDR that are observed in Africa and its rapid reversion to the wild-type strain*, we assumed that it cannot be transmitted. We investigated different impacts of NRTI-resistance on DTG-based regimen and different DTG-efficacies. For this aim, we recalibrated the model so that it reflects different odds ratios (ORs) of DTG-failure between NRTI-susceptible and NRTI-resistant individuals (OR=1, OR=2, OR=3). In the main analysis, we assumed that DTG-efficacy was similar to the one observed in the NAMSAL study, corresponding to an OR of failure between NNRTI and DTG of 1.02, after adjusting for the different baseline characteristics of the two groups (see S1 Text, Section 2.5). Other DTG efficacy corresponding to an OR of 2 and 5 were investigated in a supplementary analysis (see S1 Text, Section 5.3). All code and manuscript are available from https://github.com/anthonyhauser/MARISA2."*

3. As the manuscript describes pretreatment NNRTI resistance, and in some cases NNRTI resistance at virological failure, I would suggest the authors refrain from using the term NNRTI resistance when referring to the pretreatment time-point. Rather the authors should consider using the following terms consistently:

a. NNRTI PDR; when referring to NNRTI pretreatment resistance, and
b. NNRTI resistance; when referring to resistance at virological failure.

We corrected it throughout the manuscript, and keep "NNRTI-resistance" when we do not specifically refer to NNRTI PDR.

4. Overall, the manuscript is well written and was a pleasure to read.

Thank you.

Specific Comments

Abstract
Background

1. "We used mathematical modelling to examine the impact of the scale-up of DTG-based ART on NNRTI pre-treatment drug resistance (PDR) in South Africa, 2019-2040."
I would suggest the authors use the term "predict" rather than "examine".
In the revised version, we now use "predict" instead of "examine".

Methods and results
2. "If all men and women beyond reproductive age or on contraception are started on or switched to DTG-based ART, NNRTI resistance would reach 35.1% in 2040."
I think here the authors are trying to say, "If ONLY men and women beyond reproductive age or on contraception are started on or switched to DTG-based ART, NNRTI resistance would reach 35.1% in 2040." If so, please consider including the term only so that it distinguishes this group of people from the ones in the preceding sentence.
Thank you, we revised the sentence accordingly.

Conclusions
3. "Starting or switching all men and women to DTG would lead to a sustained decline in resistance levels whereas using DTG-based ART in all men, or in men and women beyond childbearing age, would slow down the increase in levels of NNRTI resistance."
Consider saying, "… would only slow down the increase in levels of NNRTI resistance."
We included "only" in the sentence.

Introduction
1. "Again, the rate of switching to DTG-based second-line ART will vary, influenced by concerns about the development of dolutegravir resistance in patients who switch with pre-existing resistance to NRTIs [17]." (Line 34).
The abbreviation for dolutegravir should be used as it has already been introduced previously.
Done, thank you.

2. "We adapted the MARISA model (Modelling Antiretroviral drug Resistance In South Africa) to examine the impact of different scenarios regarding the scale-up of DTG-based ART on NNRTI pre-treatment drug resistance ("NNRTI resistance" in the remaining of this article) in South Africa for 2019-2040." (Line 41).
As mentioned previously, I would suggest the authors use the term "predict" rather than "examine".
Done, thank you.

Materials and methods

3. "We added and modified parameters in order to model the introduction of DTG. We assumed that the DTG initiation rate γD→T3(t) is the same as the NNRTI initiation rate γD→T1(t) from 2019." (Line 76).
Here the assumption is that the rate at which DTG will be introduced is the same as that of NNRTIs. This assumption on the initiation rate could be limited in that less people are expected to be viraemic and to transmit HIV following the introduction of DTG, due to higher viral suppression rates with DTG compared to NNRTIs. If interpreted correctly, I would suggest the authors consider including this assumption as a limitation of the study.
It is not clear to us whether the reviewer suggests that the DTG initiation rate should be lower or higher than the NNRTI initiation rate. Nevertheless, we agree with the reviewer that many factors might influence the DTG initiation rate in the future. We already included the impact of the Treat-All policy in the model (the scenario without Treat-All policy is examined in S1 Text, Section 5.2), by increasing treatment initiation rates for people with CD4>200 copies/mL from 2017 to 2022.

Moreover, as shown by Fig. 3A, using DTG as a first-line alone has relatively little impact on NNRTI-resistance, compared with the strategies of using DTG both as first-line and switch regimen (Fig 3B). Finally, we varied switching rates to DTG over a broad range (Fig 4).

Results

4. "At the other end of the spectrum, initiating all new ART patients on DTG-based ART and rapidly switching all patients currently on NNRTI-based ART to DTG-based regimens, independently of their gender, would stabilize NNRTI resistance at a low level, with a prevalence of 14.3% (3.5%-17.5%) by 2030 and 14.8% (6.6%-19.5%) by 2040" (Line 152).
14.8% (6.6%-19.5%) NNRTI PDR with no use of NNRTIs at all still seems a bit high. Please check to make sure the model approximations are correct.
Again as mentioned in the general comments, the authors should be clear that they are referring to NNRTI PDR when they say NNRTI resistance.
Since the first submission of our paper, the model has been rewritten (to increase its computational efficiency), and it produced the same results. Therefore, we are quite confident that the model estimates are correct. However, some changes to the model (e.g. recalibrating the NNRTI suppression/failure rates) slightly decreased the estimate of NNRTI PDR, which is estimated at 8.4% in 2030 now. We comment on possible reasons why the model does not find a lower estimate of NNRTI PDR even when everybody is started on or switched to DTG in response to comment 7 of reviewer 2. To clarify this point, we added the following sentence to the Discussion:

"Finally, it is interesting to observe that, even when using DTG for everybody, NNRTI PDR still remains at a moderate level, due to the very slow reversion of NNRTI-resistance that allows subsequent transmission of NNRTI-resistance."

We agree with the reviewer that we should use "NNRTI PDR" instead of "NNRTI resistance". We adapted the manuscript accordingly.

5. "Restricting DTG-based ART to men to avoid the risk of DTG-associated neural tube defects in newborns will not curb the increase in NNRTI resistance: the prevalence of resistance is predicted to increase over the entire study period, reaching values of close to 50% by 2040."
Neural tube defects should be abbreviated as NTDs as introduced before in the introduction (Line 17). NNRTI resistance should be changed to NNRTI PDR as previously suggested, and in any instances where the authors refer to pretreatment NNRTI resistance.
Thank you. These changes have been made.

6. "As expected from their effect on NNRTI resistance, the different scenarios of the rollout of DTG-based ART also influence the rate of virological failure in women NNRTI-based ART among DTG-ineligible." (Line 181).
I think the word "on" is missing here, i.e. "…virological failure in women on NNRTI-based ART…" Besides that, this sentence is a bit confusing and the authors should consider rephrasing.
Thank you. We rephrased this sentence as follows:

"As expected from their effect on NNRTI resistance, the different scenarios of the rollout of DTG-based ART also influence the rate of NNRTI virological failure."

Discussion

7. "Overall, our findings suggest that if a large fraction of women is excluded from receiving DTG-based ART, they will not only receive a potentially inferior NNRTI-based regimen but will also

face increasing rates of resistance to this regimen due to the population level effects of continued NNRTI use." (Line 195).
This is a very important statement, but I agree with it partially. This is because levels of NNRTI PDR in women will inevitably decrease if all men are switched to DTG. So chances are that we will no longer worry about NNRTI PDR among women, especially in South Africa where it has been shown that most NNRTI PDR is due to heterosexual transmission. So the non-inferiority of the NNRTIs will play a major role in those that already got transmitted NNRTI mutations, but the effect will likely reduce drastically in later years with the reduction of NNRTI resistance among men due to DTG use. Just giving a thought.
We thank the reviewer for raising this point because the aim of this study was precisely to question the intuitive idea that a wide introduction of DTG might lead to a drastic decrease in NNRTI resistance in the long term. The model shows that, even when everybody is started on or switched to DTG-based regimen (red line in Fig 3B/C), the level of NNRTI-resistance stabilizes to values between 10%-15% and does not substantially decrease. This is mainly due to the low fitness cost of NNRTI resistance, which prevents resistant strains to revert back to wild-type virus. As a consequence of the low reversion rate [3], individuals infected with an NNRTI-resistant strain might then transmit NNRTI-resistance to other individuals [4].

8. "Model simulations emphasize the importance starting on or switching a maximum number of women to…"
I think the sentence is missing the word "of", i.e. "Model simulations emphasize the importance of starting on or switching a maximum number of women to…"
Thank you, corrected.

Reviewer #3
This is a modelling study concerned with an interesting issue, upscaling the use of Dolutegravir. While the paper is well-written and carefully conducted, some methodological issues related to the treatment of uncertainty are unclear and require clarification. Specific comments are given below.

This article is essentially a large extrapolation exercise into the future. Extrapolations are potentially dangerous and should be made with caution, placing particular attention to uncertainty representation. Here the authors conduct a multivariate (multi-way) sensitivity analysis and this is appropriate. In addition, they depict uncertainty in their figures in a clever way, thus avoiding cluttering in the main part of the figure.
However, the analysis is based on deterministic models which, depending on their treatment, tend to underestimate uncertainty. To this end it would be useful to add the evolution of uncertainty over time.
Thank you. We added Fig 3 to S1 Text showing the evolution of uncertainty over time for the 13 scenarios.

Also, it would be useful to report the uncertainty of each parameter used. For example, on table 1 and table 2 of the supplement they could add the parameter value, it's range and any distributional assumptions made for each parameter. For the calibration parameters (supplement table 1) this is vital since the results of any survival analysis should incorporate the associated uncertainty.
Tables 1 and 2 have been modified to include the 95% CI of the estimates. Rates were estimated using survival analysis. More information about the method can be found in [5].

This issue also relates to the selected range of the sensitivity analysis, how were the numbers on supplement table 4 informed, were they evidence-based or assumption-based?

In the sensitivity analysis, we selected the parameters that have a direct impact on the transmission of NNRTI-resistance, i.e. parameters related either to HIV-transmission or to NNRTI-resistance. The lower and upper bounds of these parameters, shown in Table 4 of S1 Text, are assumption-based. We chose wide parameter ranges to reflect the uncertainty related to NNRTI-resistance and to show the difficulty of predicting the level of NNRTI-resistance over the next 20 years. The aim of the study is not to provide an estimate of NNRTI-resistance levels in 2040, but to compare the relative impact on NNRTI TDR of different strategies of DTG-introduction.

Finally, it would be good to partly amend the discussion of the results, fully reflecting the uncertainty of the findings, including the absence of "statistically significantly different" results, which simply give a scientifically honest picture reflecting the inherent uncertainty of such modelling exercises.
We agree with the reviewer that model-based estimates are subject to a high level of uncertainty and now acknowledge this fact as follows:

"Second, predictions of levels of NNRTI resistance over the next twenty years are naturally uncertain, as reflected by the wide sensitivity ranges in Fig 3."

It is, however, essential to note that overlapping confidence interval, as in Fig 3, does not mean that the two scenarios are not "statistically different", as the different scenarios are not independent. For example, increasing the NNRTI resistance acquisition rate, which is one of the eight parameters included in the sensitivity analysis, will increase the level of NNRTI resistance in all scenarios. To better visualize the difference in scenarios, we added Fig 4 to S1 Text. It shows the difference in NNRTI TDR between the different scenarios of DTG-introduction and the scenarios where DTG is not introduced. We observe that for all scenarios, the upper bound of the 95% sensitivity range is below 0 in 2040, showing "statistical difference" between the scenarios with and without DTG-introduction.

The design of this modelling study crucially depends upon the selected scenarios. One plausible option is to look extensively into the consequences of using NNRTI-based ART as first line treatment and DTG-based ART as second line. Presumably the evidence and long-term knowledge of the genetic interactions this may cause and the possibility of future DTG resistance development is relatively scarce? It appears that these issues deserve further discussion.
We did not model the scenario where DTG-based ART is only used as a second-line regimen. This would be at odds with the guidelines [6]. South Africa is already using DTG-based for ART-naïve individuals. The impact of including DTG in second-line ART can nevertheless be observed in Fig 3 (when comparing Fig 3A with Fig 3B/C) and in Fig 4, where we allow different switching rates to DTG.

References

[1] Rhee S-Y, Grant PM, Tzou PL, Barrow G, Harrigan PR, Ioannidis JPA, et al. A systematic review of the genetic mechanisms of dolutegravir resistance. J Antimicrob Chemother 2019;74:3135–49. doi:10.1093/jac/dkz256.

[2] Moorhouse M, Maartens G, Venter WDF, Moosa M-Y, Steegen K, Jamaloodien K, et al. Third-Line Antiretroviral Therapy Program in the South African Public Sector: Cohort Description and Virological Outcomes. J Acquir Immune Defic Syndr 2019;80:73–8. doi:10.1097/QAI.0000000000001883.

[3] Yang W-L, Kouyos RD, Böni J, Yerly S, Klimkait T, Aubert V, et al. Persistence of Transmitted HIV-1 Drug Resistance Mutations Associated with Fitness Costs and Viral Genetic Backgrounds. PLOS Pathog 2015;11:e1004722. doi:10.1371/journal.ppat.1004722.

[4] Yang, Wan-Lin and Kouyos, Roger and Scherrer, Alexandra U. and Böni, Jürg and Shah, Cyril and Yerly, Sabine and Klimkait, Thomas and Aubert, Vincent and Furrer, Hansjakob and Battegay, Manuel and Cavassini, Matthias and Bernasconi, Enos and Vernazza, Piet for the SHCS. Assessing the Paradox Between Transmitted and Acquired HIV Type 1 Drug Resistance Mutations in the Swiss HIV Cohort Study From 1998 to 2012. J Infect Dis 2015;212:28–38.

[5] Hauser A, Kusejko K, Johnson LF, Wandeler G, Riou J, Goldstein F, et al. Bridging the gap between HIV epidemiology and antiretroviral resistance evolution: Modelling the spread of resistance in South Africa. PLOS Comput Biol 2019;15:e1007083. doi:10.1371/journal.pcbi.1007083.

[6] Republic of South Africa National Department of Health. 2019 ART Clinical Guidelines for the Management of HIV in Adults, Pregnancy, Adolescents, Children, Infants and Neonates. 2020.
